# Fathers favour sons, mothers don't discriminate: Sex-biased parental care in northwestern Tanzania

**DOI:** 10.1017/ehs.2019.14

**Published:** 2019-12-04

**Authors:** Anushé Hassan, Susan B. Schaffnit, Rebecca Sear, Mark Urassa, David W. Lawson

**Affiliations:** 1 Department of Population Health, London School of Hygiene and Tropical Medicine, Keppel Street WC1E 7HT, UK; 2 Department of Anthropology, University of California, Santa Barbara, CA 93106, USA; 3 National Institute of Medical Research, Mwanza, Tanzania

**Keywords:** parental investment, sex-biased care, paternal son-bias, early childhood, Tanzania

## Abstract

Variation in parental care by child's sex is evident across cultures. Evolutionary theory provides a functional explanation for this phenomenon, predicting that parents will favour specific children if this results in greater fitness payoffs. Here, we explore evidence for sex-biased parental care in a high-fertility, patriarchal and polygynous population in Tanzania, predicting that both mothers and fathers will favour sons in this cultural setting. Our data come from a cross-sectional study in rural northwestern Tanzania, which included surveys with mothers/guardians of 808 children under age 5. We focus on early childhood, a period with high mortality risk which is fundamental in establishing later-life physical and cognitive development. Examining multiple measures of direct/physical care provision (washing, feeding, playing with, supervising, co-sleeping and caring when sick), we demonstrate that fathers favour sons for washing, feeding and supervising, while maternal care is both more intensive and unrelated to child sex. We find no difference in parental care between girls and boys regarding the allocation of material resources and the duration of breastfeeding; or in terms of parental marital and co-residence status. This bias towards sons may result from higher returns to investment for fathers than mothers, and local gender norms about physical care provision.

## Graphical abstract

**Figure:**
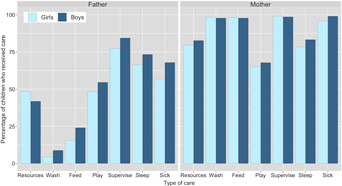

**Media summary:** Fathers provide more direct care to sons, while mothers care for sons and daughters equally in northwestern Tanzania.

## 1. Introduction

A broad principle of parental investment theory posits that natural selection will favour equal parental care for sons and daughters if rearing both sexes is equally costly, as each sex provides exactly half the genes for all future descendants (Fisher 1930). However, the costs and benefits of investment in each sex are rarely uniform (Hamilton 1967; Trivers and Willard 1973), and discriminatory parental care by offspring sex is observed across human cultures. Parental investment is defined as any allocation of resources which benefits offspring at a cost to a parent's ability to invest in other components of fitness, while parental care more broadly refers to any parental trait that enhances the fitness of offspring, and is likely to have originated and/or to be maintained for that function, without necessarily being costly to the parent (Royle *et al*. 2012; Trivers, 1972). Parental care is the more appropriate term when costs to parental fitness are not directly estimated. The focus of this paper is on post-natal parental care, as opposed to biases in sex ratio at birth. Sex-biases in post-natal care may include such factors as discriminatory feeding, supervision, expenditure on health care and schooling, along with differential allocation of resources throughout life, including the transfer of inheritance.

When sex-biased parental care is observed it is most commonly biased in favour of sons (Hartung *et al*., 1976; Khera *et al*. 2014; Williamson, 1976). Son-preference is perhaps most evident in some East and South Asian societies (Das Gupta *et al*. 2003; Murphy *et al*. 2011), but has also been widely reported in sub-Saharan Africa (Campbell, 1991; Fayehun *et al*., 1997; Frempong and Codjoe 2017). Parental biases favouring sons will be adaptive when the marginal returns to investing in sons is greater than for daughters (Keller *et al*. 2001; Ross *et al*. 2016; Veller *et al*. 2016). This scenario may especially characterize contexts where variability in male fitness is extended via polygynous marriage so that successful males obtain particularly high reproductive success (Clutton-Brock *et al*. 1981; Leimar, 1996; Irwin *et al.* 2006; but see Brown *et al*. 2009). From a proximate economic viewpoint, investing in a son may also maximise chances of future financial and social returns and support in old age if men are valued over women for providing family labour and financial security for parents throughout their life-course (Becker and Tomes, 1976; Mutharayappa, 1997).

On the other hand, in some populations, parents invest more in daughters. This has been recorded, for example, among the Mukogodo of Kenya (Cronk, 1989, 1991b), and the Mosuo of China (He *et al*. 2016). One hypothesis suggested by researchers to explain daughter-preference is the concept of ‘local resource enhancement’ or ‘helpers at the nest’ (Pen and Weissing 2000; Quinlan and Quinlan 2005). This hypothesis posits that a disparity in the productivity of boys and girls as helpers in the household may bias favour towards the more helpful sex when that family does not have a sufficient number of that sex, whether male or female (Quinlan and Quinlan 2005). In societies that favour daughters, girls tend to partake more than boys in activities that benefit the family economically and/or help more with housework and caring for younger children (Bereczkei and Dunbar, 1997, 2002; Hames and Draper 2004; Margulis *et al*. 1993). Daughter-biased investment has been recorded among multiple populations, including American Hutterites (Margulis *et al*., 1993), communities in Tibet and China (Childs *et al*. 2011; Du and Mace 2017; Zhan and Montgomery 2003) as well as the !Kung in Botswana (Hames and Draper 2004).

Complicating the study of parental care, previous studies often quantify discriminatory treatment of sons and daughters using measures that may not accurately reflect parental intentions or capture actual parental behaviour. Such measures include self-reported preferences of parents (Brunson 2010; Cronk, 1991a; Du and Mace 2017); child outcomes such as health and mortality as proxies for differential investment (Arnold *et al*. 1998; Chen *et al*. 1981; Klasen, 1996; Svedberg, 1990); along with skewed sex ratios at birth and/or other ages (Guilmoto 2012, 2015). These measures may be problematic for a number of reasons. First, there are often discrepancies between stated sex preferences and who parents actually invest in: one study in Amdo Tibet found girls were favoured due to their increasing economic value in a community where stated cultural norms favour males (Du and Mace 2017); and similar discrepancies have been documented among the Mukogodo in Kenya, where there is a dissonance between stated cultural norms, which favour boys, and parental behaviour which is daughter-biased (Cronk 1991a). Second, using differences in the wellbeing or survival of males and females to infer differences in care is problematic because such measures can vary independently of parental care in non-trivial ways. Male and female developmental trajectories are distinct, and males are generally subject to higher neonatal and infant mortality than females independently of parental behaviour (Wells 2000). Likewise, educational attainment is now higher for females in most high-income populations, but this may reflect male vulnerabilities to mental health issues or other factors which favour school dropout (e.g. incarceration) rather than higher parental investment in daughters (Grant and Behrman 2010; McDaniel 2012). Finally, it is important to note that natural selection is anticipated to act independently on sex-ratio biasing and post-natal investments (Veller *et al*. 2016), so that evidence of one (e.g. a male biased sex ratio) should not be taken as evidence of the other (e.g. indication that male offspring are treated differently by parents after birth).

Quantifying differences in parental behaviour is thus preferable, especially behaviours most likely to be both costly to parents and beneficial to offspring (and so fitting the formal definition of parental investment; Clutton Brock 1991; Royle *et al*. 2012). Such measures can include conspicuous transfers of capital (e.g. at inheritance; Hartung *et al*. 1976; Hrdy and Judge 1993) and observations or reports of provisioning that requires physical proximity and/or possibly energetic expenditure on the part of the carer (Baker and Milligan 2016; Bereczkei and Dunbar 1997; Cronk 1991b; Lawson and Mace 2009; Nikiforidis *et al*. 2018). In this paper, we explore evidence of sex-bias in post-natal parental care in a rural northwestern Tanzanian population. We focus on children under 5 years because providing adequate care at this age is crucial for child health (WHO 2018). Children are vulnerable during this period, experiencing a high rate of preventable mortality [41 deaths per 1000 live births globally in 2016 (WHO 2017)]. Additionally, this life-stage sets future trajectories of child growth; among other complications, poor feeding practices and malnutrition can result in stunting, wasting, underweight or overweight and obesity, which may have health implications throughout the life-course (Almond and Currie 2011; Maluccio *et al*. 2009; Palloni 2017). We consider four dimensions of parental care, measured through behaviour reported by children's mothers or guardians: (a) allocation of material resources, which we classify as indirect care provision as this can take place without any interaction between the child and carer; (b) provisioning that requires the carer to expend energy, interact with the child or be in physical proximity to the child (washing, feeding, playing with, supervising, co-sleeping and caring for when sick), which we classify as direct/physical care provision; (c) breastfeeding duration, a well-established determinant of child survival and nutrition outcomes (Lawson *et al*. 2012; Sellen 2007); and (d) parental marital status and co-residence, which we treat as a commitment to parental care, especially from fathers (see Dahl and Moretti 2008).

In the study population, girls play a valuable role in contributing to household work (Hedges *et al*. 2018) and bridewealth is commonly practised (Schaffnit *et al*. 2019a) indicating that daughters may perhaps be energetically and financially beneficial for parents. However, high levels of fertility and polygynous marriage in the northwestern regions of the country are suggestive of both higher variation in male than female reproductive success and more opportunities for men to translate invested resources into reproductive success. In 2016, in Tanzania's Lake Zone (where our study was conducted) the total fertility rate was 6.4 births per woman; and 22.2% of married women stated having at least one co-wife (Ministry of Health Gender, Elderly and Children - MoHCDGEC/Tanzania Mainland *et al.*, 2016). Additionally, substantial value is placed on men in many Tanzanian communities, visible in traditionally practised patrilineal systems of marriage and wealth inheritance among local peoples, e.g. marital systems are usually extended patrilocal, with women moving into their husbands’ households after marriage, and wealth and land are most often passed primarily from father to son (Ezer 2002). Investment biases favouring sons are usually present in such contexts, especially where polygynous marriage is common (Das Gupta *et al*. 2003; Hartung *et al*. 1976; Mace 1996; Williamson 1976). Therefore, we expect that parents will bias care towards their sons across all measures.

Our study has two major strengths. First, we consider a wide range of measures of parental care within the same population. Second, we explore provision of care from both mothers and fathers. Most studies of sex-biased care focus either on mothers or investment from both parents, neglecting the role of fathers even though parental behaviour (and the subsequent fitness returns to investment) may vary by *both* the child's and the parent's sex (as documented in some high-income populations; Lawson and Mace 2009; Nettle 2008; Nikiforidis *et al*. 2018).

## 2. Data and methods

### 2.1. Data collection

Our data come from two rural communities (one rural but rapidly urbanizing town and one rural village) in northwestern Tanzania situated within the bounds of the Magu Health and Demographic Surveillance Site, which has been active in the area since 1994 (Kishamawe *et al.* 2015; see also Hedges *et al.* 2018). The area is primarily Sukuma. Although Tanzania is home to considerable ethnic diversity, the Sukuma are the largest ethnic group in the country, comprising approximately 17% of the national population (Malipula 2016). We randomly sampled 743 households to meet the requirements of a larger project studying the wellbeing of women aged 15–35 years and their children (see Schaffnit *et al*. 2019b). The data used for this paper comes from surveys conducted in the 506 households that had a resident child aged under 5 years, with 808 children surveyed. Each household survey recorded household membership, size and composition, and the demographic and socio-economic characteristics of the household head and all household members, including members’ relationship to household head, household food insecurity and land ownership. All indicators used in this paper that pertain to the child and the child's parents were then measured via a child survey. The child survey was directed to either the child's biological mother or to the primary guardian if the mother was unavailable (with 87% of surveys being completed by the child's biological mother). The subsequent respondent answered all questions on the survey, including those about behaviours (i.e. care and resource provisioning) from other relatives, including both biological parents. All interviews were carried out in Swahili or Sukuma using Open Data Kit Collect software on electronic devices. Ethical approval was granted by LSHTM (13809), UCSB (1-17-0405) and NIMR (MR/53/100/463).

### 2.2. Variables used and data analysis

Parental care was measured across several dimensions (our dependent variables) and associations with sex of the recipient child (the primary independent variable) were analysed using logistic regression and survival analysis depending on the measure of care (see below). Treating the child's sex as an exogenous variable (i.e. there are likely to be few confounders of the associations we test), in all models, we adjusted only for child's age (continuous measure) and age-squared. We did not run multi-level models as we surveyed an average of 1.75 children per household and research shows that fixed and random effects may both be overestimated in two-level models when clusters are unbalanced and observations per group are sparse, i.e. fewer than two observations per group (Clarke 2008). We acknowledge that this may result in standard errors being biased downwards.

Allocation of material resources was captured in a binary variable indicating whether the child had received resources from mothers and fathers (whether co-resident or non-co-resident with the child) in the 3 months preceding the survey (mothers, *n* = 807, 1 refusal; fathers, *n* = 807, 1 ‘don't know’). Resources could include food, medicine, clothes, money, household goods or ‘other’. Direct/physical care provision was captured in six binary variables (*n* = 808 for both parents unless stated otherwise) indicating whether mothers and fathers had washed, fed or cooked for, played with, supervised or monitored, slept in the same room as the child (mothers, *n* = 807, 1 missing) or cared for the child if sick in the 2 weeks preceding the survey (215 children had been sick in this time period: 103 girls and 112 boys; *n* = 215 for both parents). Children whose mothers or fathers were not alive at the time of survey (mothers, *n* = 6; fathers, *n* = 9) were excluded from the analysis. Logistic regression models were used to test for associations between each measure of parental care and child's sex.

Mothers’ investment in breastfeeding was measured in two ways. Firstly, for children who had stopped breastfeeding, we asked the respondent to report on time spent exclusively breastfeeding (i.e. a time period during which the child was given no other drink or food apart from breastmilk). A binary variable indicated exclusive breastfeeding for ‘less than 6 months’ or ‘6 or more months’ (*n* = 541; excluded: 5 children whose mothers had died, 5 who had never been breastfed, an additional 3 who had never been exclusively breastfed, 14 for whom the respondents did not know if they had ever been exclusively breastfed and the 240 babies who were still breastfeeding at time of survey). Secondly, for all children, we asked the respondent what age the child had stopped breastfeeding completely. Child's age at breastfeeding termination was measured in months and coded as a continuous variable (*n* = 798; excluded: 5 children who had never been breastfed and 5 whose mothers had died; all non-resident mothers (*n* = 74) had breastfed their children so were included in the analysis). The 240 children still breastfeeding at time of survey were included in the analysis as right-censored cases (see below).

A logistic regression model was used to explore whether girls had higher odds of terminating exclusive breastfeeding before 6 months. Discrete-time event history analysis was used to test for an effect of child's sex on duration of overall breastfeeding: heaping of events at ages 6, 12 and 18 months meant that discrete-time survival analysis was the most appropriate method to use.

Two indicators measured parental relationship status. The first was whether the child's parents were married or divorced, regardless of co-residence or marital type (i.e. polygynous or monogamous). This included only those children whose parents were currently married (*n* = 555) and those whose parents were separated or divorced (*n* = 98), with a total sample of 653 children. Children were excluded if the respondent did not know (*n* = 1) or refused to answer (*n* = 1); if one or both parents were not alive (*n* = 14); or if the parents were not in a relationship during the survey period and had never married and those who were in a relationship but unmarried. Secondly, parental relationship status was measured as whether the child's parents co-resided or not, regardless of marital status (*n* = 793; excluded: if one or both parents not alive, *n* = 14; refusal, *n* = 1). We acknowledge that parents’ relationship status can be contingent on a complicated decision-making process, which may not always (or entirely) reflect investment in children, and is thus not an obvious or refined measure of parental care. However, we believe it can still provide important information about parenting in our study population.

We fit multivariate logistic regressions to examine the association between child's sex and parental marital status or co-residence. Considering we do not have data on children's elder siblings, whose sex may impact parental relationships, we also ran a sensitivity analysis restricting our sample to only first children of parents (*n* = 101 for marital status and *n* = 166 for co-residence).

## 3. Results

### 3.1. Household and child characteristics

There was an average of 7.7 household members and 1.7 children under age 5 years resident in each of the 506 households containing at least one child (Table 1). The majority of households were of Sukuma ethnicity (90%), identified with a form of Christianity (Roman Catholic, 36%; other Christian, 36%) and had a male household-head (81%). Most households-heads were educated to primary level (66%) with very few having progressed further (11%) and the remaining had no education (22%; don't know = 1%). A little more than half of the household-heads listed farming as their main occupation (55%), followed by trading (21%). A large percentage of households scored high on food insecurity; 57% were categorised as severely insecure and 21% as moderately insecure. Food insecurity was measured using the Household Food Insecurity (Access) Scale (Coates *et al*. 2007), which records whether the household experienced problems with accessing food in the past month.

**Table 1.** Household- and child-level characteristics

	Girls	Boys	Total
Number of households with children 0–5 years			506
Number of total children 0–5 years	397	411	808
*Household characteristics*			
Household size – mean (min, max)			7.67 (3, 25)
Number of children aged 0–5 in household – mean (min, max)			1.75 (1, 7)
Food insecurity – *n* (%)			
Food secure			94 (18.61)
Mildly food insecure			19 (3.76)
Moderately food insecure			106 (20.99)
Severely food insecure			286 (56.63)
*Child characteristics*			
Age continuous – mean (min, max)	2.44 (0, 5)	2.42 (0, 5)	2.43 (0, 5)
Age in years – *n* (%)			
0–1 years	76 (19.14)	83 (20.19)	159 (19.68)
1–2 years	78 (19.65)	78 (18.98)	156 (19.31)
2–3 years	81 (20.40)	85 (20.68)	166 (20.54)
3–4 years	94 (23.68)	90 (21.90)	184 (22.77)
4–5 years	68 (17.13)	75 (18.25)	143 (17.70)
First child of biological father – n (%)			
Yes	89 (23.06)	78 (19.65)	167 (21.33)
No	291 (75.39)	314 (79.09)	605 (77.27)
Don't know	6 (1.55)	5 (1.26)	11 (1.40)
Breastfeeding duration^a^ – n (%)			
0–5 months	11 (4.01)	6 (2.11)	17 (3.05)
6–11 months	18 (6.57)	19 (6.69)	37 (6.63)
12–17 months	113 (41.24)	132 (46.48)	245 (43.91)
18–23 months	83 (30.29)	83 (29.23)	166 (29.75)
23–26 months	49 (17.88)	44 (15.49)	93 (16.67)
*Parent characteristics*			
Mother's residence/death – *n* (%)			
Lives in household	361 (90.93)	367 (89.29)	728 (90.10)
Does not live in household	32 (8.06)	42 (10.22)	74 (9.16)
Dead	4 (1.01)	2 (0.49)	6 (0.74)
Father's residence/death – *n* (%)			
In the household	265 (66.75)	282 (68.61)	547 (67.70)
Not in the household	123 (30.98)	117 (28.47)	240 (29.70)
Dead	4 (1.01)	5 (1.22)	9 (1.11)
Don't know/refusal	5 (1.26)	7 (1.70)	12 (1.49)
Parents’ marital status – *n* (%)			
Married	275 (71.24)	280 (70.53)	555 (70.88)
Not married	110 (28.50)	116 (29.22)	226 (28.86)
Don't know/refusal	1 (0.26)	1 (0.25)	2 (0.26)

^a^ Among weaned children only (*n* = 558).

An equal proportion of girls and boys were surveyed with ages ranging from 7 days old up to 5 years. Whereas almost all children resided with their biological mothers (90%), one-third did not live with their biological fathers (of those with a living father). Almost one-third of children's biological parents were not married to each other, and the most common reason for this was separation or divorce.

### 3.2. Resource allocation and direct/physical care provision

**Figure 1.**
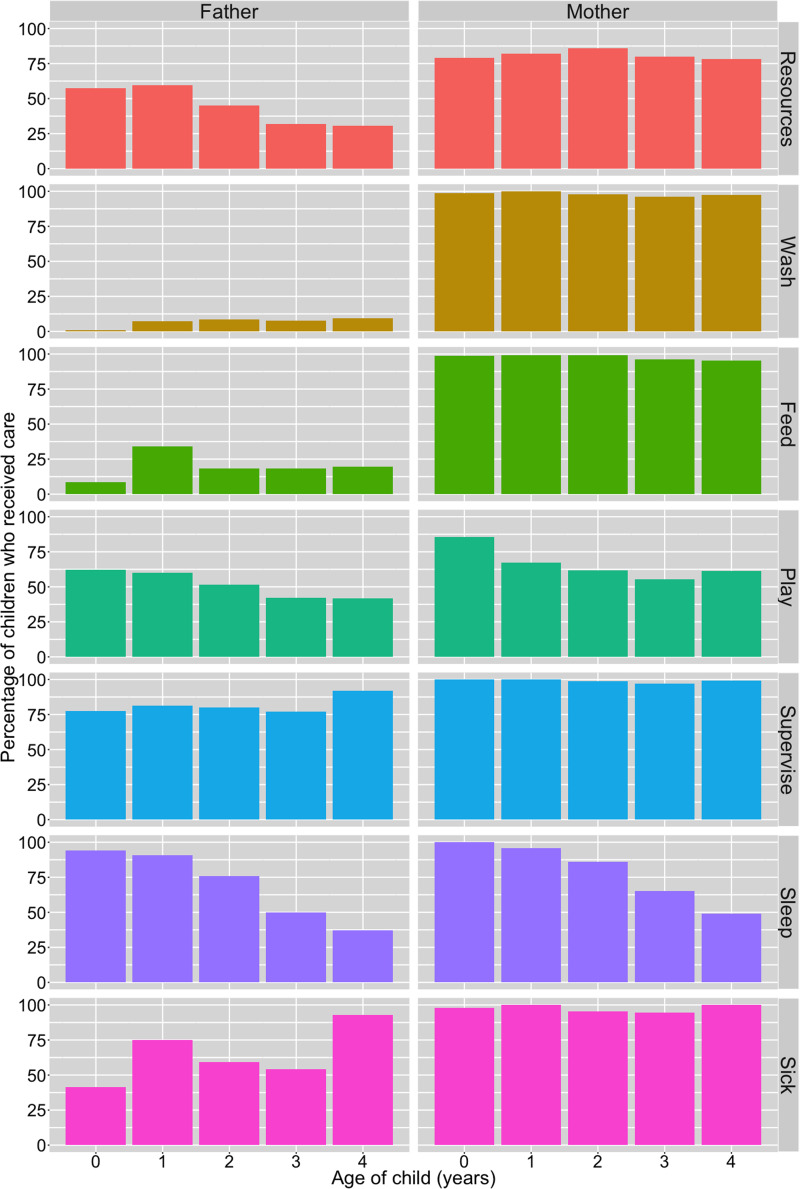
Percentage of children reported to receive material resources in past 3 months and direct/physical care in past 2 weeks from their biological fathers and mothers, by child's age (years). Resource provision is from alive mothers (*n* = 801; excluded ‘refusal’ *n* = 1) and non-co-resident fathers (*n* = 239; excluded ‘don't know’ *n* = 1); direct care is from co-resident parents only (mothers, *n* = 728; fathers, *n* = 547); caring for sick children limited to children who had been sick in past 2 weeks (*n* = 215).

A breakdown of resource and direct/physical care provision by child's age and parent's gender is presented in Figure 1. Of the total sample of children, a majority were reported to have received resources from their mothers and fathers in the 3 months preceding the survey, and both parents were equally likely to have provided resources in this time period (81% from mothers; 81% from fathers). However, resource provision from fathers differed by paternal residence: 99% of co-resident fathers (*n* = 547; 68% of total sample) had supported their child by providing resources. In contrast, among non-co-resident fathers (*n* = 240; 30%) only 45% had provided resources in the past 3 months. Owing to the lack of variation in resource provisioning by fathers among children with co-resident fathers, we restricted analyses regarding resource provision from fathers to children with non-co-resident fathers only. There was no evidence of a difference between resource provision to boys and girls from either parent (Table 2; Supplementary Tables S2.1 and S2.2).

**Table 2.** Logistic regression outputs showing associations between child's sex and each type of parental care provision. Effect sizes (odds ratios) adjusted for child's age (continuous) and age-squared. Full models for each type of care available in Supplementary Material Tables S2.1–S6.4. Resource allocation is from alive mothers (*n* = 801) and non-resident fathers (*n* = 239); all six forms of direct/physical care are from co-resident parents only (mothers, *n* = 728; fathers, *n* = 547); caring for sick children is limited to children who had been sick in past 2 weeks (*n* = 215)

	Odds ratio (95% CI)							
**Type of care**	**Resource allocation**		**Washing**		**Feeding**		**Playing**	
	Mothers	Fathers	Mothers	Fathers	Mothers	Fathers	Mothers	Fathers
*n*	807	239	728	547	728	547	728	547
Child is male	1.21 (0.84*–*1.73)	0.86 (0.51*–*1.46)	0.75 (0.26*–*2.19)	2.19* (1.07*–*4.47)	0.88 (0.31*–*2.46)	1.76** (1.14*–*2.71)	1.12 (0.81*–*1.53)	1.24 (0.88*–*1.74)
**Type of care**	**Supervising**		**Sleeping next to**		**Caring when sick**		**Exclusive breastfeeding**	
	Mothers	Fathers	Mothers	Fathers	Mothers	Fathers	Mothers	
*n*	728	547	727	547	204	143	541	
Child is male	0.59 (0.14*–*2.48)	1.63* (1.06*–*2.52)	1.45† (0.95*–*2.20)	1.28 (0.84*–*1.93)	4.30 (0.47*–*39.47)	1.56 (0.78*–*3.1)	0.85 (0.60*–*1.20)	
**Type of care**	**Parents married vs divorced**		**Parents married vs divorced**		**Parents’ co-reside vs live apart**		**Parents’ co-reside vs live apart**	
	Full Sample		First Child Only		Full Sample		First Child Only	
*n*	653		101		793		166	
Child is male	1.00 (0.65*–*1.55)		1.13 (0.45*–*2.82)		1.12 (0.83*–*1.51)		1.42 (0.74*–*2.73)	

^a^Among weaned children only (*n* = 558).

With regards to direct/physical care, survey respondents stated that mothers provided all six types more often than fathers in the 2 weeks preceding the survey (Figure 1). Very few non-co-resident mothers and fathers were reported as providing any of the six types of this care to their children during this time period and so we excluded these parents from our analysis: non-co-resident mothers – washing (*n* = 2, 3%), feeding (*n* = 5, 7%), playing with (*n* = 2, 3%), supervising (*n* = 4, 5%), co-sleeping (*n* = 2, 3%) and caring for when sick (*n* = 1, 10%); non-co-resident fathers – washing (*n* = 0), feeding (*n* = 8, 3%), playing with (*n* = 19, 8%), supervising (*n* = 18, 8%), co-sleeping (*n* = 11, 5%) and caring for when sick (*n* = 7, 10%).

**Figure 2.**
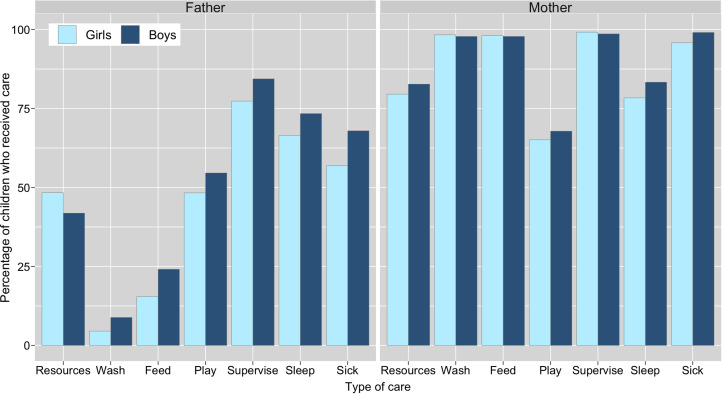
Percentage of children reported to receive material resources in past 3 months and direct/physical care in past 2 weeks from their biological fathers and mothers, by child's sex. Resource provision is from alive mothers (*n* = 801; excluded: ‘refusal’, *n* = 1) and non-co-resident fathers (*n* = 239; excluded: ‘don't know’, *n* = 1); direct care is from co-resident parents only (mothers, *n* = 728; fathers, *n* = 547); caring for sick children limited to children who had been sick in past 2 weeks (*n* = 215). Logistic regression analyses show evidence of a difference in care provision by child's sex (for washing, feeding and supervising) from fathers, but not mothers. Odds ratios for each type of care are shown in Table 2.

A greater percentage of boys than girls were reported to have received all types of direct/physical care from their co-resident fathers, whereas the results from co-resident mothers were inconsistent, with little visible difference in care provision between sons and daughters (Figure 2). Logistic regression models showed no difference between boys and girls for any of the six types of direct/physical care provision from co-resident mothers: confidence intervals for odds ratios crossed 1 and *p*-values were >0.1 (Table 2; Supplementary Figure S1; Supplementary Tables S3.1–S3.6). Sons had higher odds of receiving all six types of direct/physical care from co-resident fathers than daughters, with strong evidence of a difference in odds (at *p* < 0.05) for washing, feeding and supervising the child (Table 2). For the other activities, effect sizes were comparable but in all cases 95% confidence intervals crossed 1 and *p*-values were >0.1 (Table 2; Supplementary Figure S1; Supplementary Tables S4.1–S4.6).

### 3.3. Breastfeeding duration, parental marital status and co-residence

**Figure 3.**
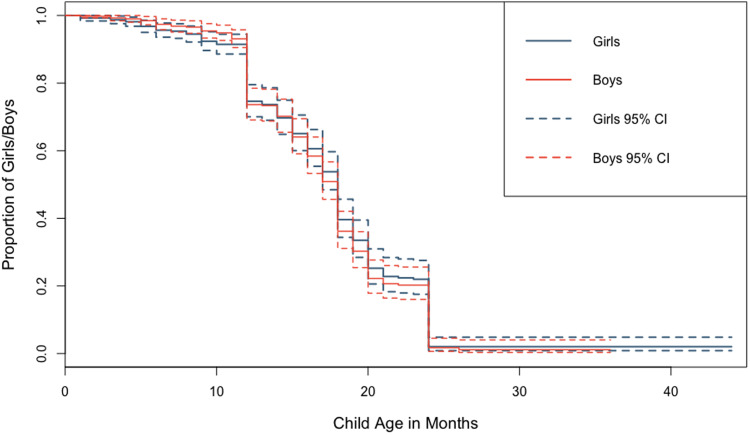
Kaplan–Meier survival curves showing difference in overall breastfeeding duration between boys and girls with 95% confidence intervals.

There was almost universal coverage of breastfeeding among the children surveyed (99% of children experienced at least some breastfeeding), with 30% of children still breastfeeding during the survey period. Among weaned children (*n* = 558), the median time to weaning was 17 months; this did not differ by child's sex. The majority of weaned children were breastfed exclusively for at least 6 months (62%). More girls were reported as being exclusively breastfed for at least 6 months (63%) than boys (60%), but this small difference was not statistically significant (Supplementary Table S5.1). A Kaplan–Meier survival curve showed no visible difference between duration of overall breastfeeding between sons and daughters and a log-rank test conducted to check equality of the survivor function across both sexes confirmed this (*p* = 0.27). Discrete-time survival analysis showed no difference in age at weaning among sons and daughters (Figure 3; Supplementary Tables S5.2–S5.3; Supplementary Figure S2). Neither parental marital status nor residential situation were related to children's sex (Table 2; Supplementary Tables S6.1–S6.4).

## 4. Discussion

Sex-biased parental care is common throughout the world with parents expected to direct investment towards the sex with a higher fitness payoff. In this rural Tanzanian context, analysing reports of parental care behaviour from children's mothers or guardians, we find that fathers favour sons in several measures of direct/physical parental care, but mothers do not discriminate their care in any form – resource provisioning, direct/physical care, or breastfeeding duration – based on their child's sex.

We explored if mothers and fathers provided care differentially to children, without making *a priori* predictions about whether or how sex-bias would vary between them. Previous research suggests that mothers and fathers can differ in their sex-preference for children as well as in thecare they give to sons and daughters. For example, patterns similar to our finding that fathers favour sons but mothers don't discriminate have been seen in both contemporary high-income settings like the US(preference for male children among fathers, Newport 2018; son-biased paternal involvement, Harris *et al*. 1998) and in another Tanzanian population (among Hadza hunter-gatherers, Marlowe 2003). Other studies document a paternal bias towards sons without reporting on maternal biases. For example, researching men's preferences for their children's sex and resultant contraceptive behaviour, Mwageni *et al*. find men to have a strong inclination towards having sons over daughters (Mwageni *et al*. 2001), and Nettle finds that fathers invest more in sons vs daughters among a large British cohort (Nettle 2008). Research also reports on maternal biases towards daughters without collecting data on fathers (Suitor and Pillemer 2006). One particularly large-sample study of British families finds that fathers spend more time engaging in childcare activities with sons while mothers favour daughters (Lawson and Mace 2009). Analysing data from South Africa, Bangladesh, Indonesia and Ethiopia, one study finds substantial variation by country in parental investment in children's education by both child and parent's gender (Quisumbing and Maluccio 2003). The authors highlight the need to consider context-specific factors that drive parental gender preferences. A recent study of parental time investment among East and South Asian families in the US (more than nine countries of origin included) suggests that norms of son preference persist post-migration but only for mothers (Kaushal and Muchomba 2018). Here, mothers spend more time with young sons than daughters whereas fathers are gender neutral with this age-group (0–5 years); as children grow older, mothers spend more time with daughters and fathers with sons (6–17 years).

What lies behind such variation in the behaviour of mothers vs. fathers in relation to child sex is not immediately obvious, but may reflect contextual differences in sex-specific costs and benefits of care and/or related cultural variation in gendered division of parenting. One explanation for fathers caring more for boys than girls in the context of rural Tanzania could be that fitness interests of fathers and sons are more closely associated than those of fathers and daughters, resulting in greater investment from fathers in sons. For example, in patrilineal and patrilocal societal structures male relatives may cooperate more with each other as residential and descent patterns favour men, whereas women move away from their relatives and do not inherit either the family name or wealth (Gibson 2008; Pashos and Mcburney 2008). Mothers on the other hand may invest equally because they stand to receive equal returns from both sexes: as well as receiving the benefits sons are expected to bring in terms of reproductive and financial payoffs, they also benefit from the help daughters provide with housework and childcare later in life (which may have relatively little impact on fathers). It would be instructive to explore this possibility with data on the long-term consequences of parental investment in sons versus daughters.

It is also possible that the patterns we observe are not adaptive or meaningful from a fitness perspective but nevertheless in line with local cultural customs. On a proximate level, our findings are consistent with articulated gender norms relating to parental care in Kisesa. In exploratory focus group discussions with parents of children under 5 years of age (conducted alongside quantitative data collection), both mothers and fathers commented on gendered aspects of parenting. Several mothers indicated that direct physical care of daughters by fathers was taboo, with one stating ‘he can help you wash and clothe the child, but it should not be a female child … it's normal for a man to wash a male child but not a female child’ and another corroborating this ‘when a female child reaches two or three years old she shouldn't be washed by her father’. This sentiment was echoed by fathers, with one stating ‘I think the girl child under the age of five, may be some are afraid of female gender … people here are sensitive with gender … the big percent is done by women’. While not all parents shared these views (one parent countered that child sex was of little relevance ‘the issue is not whether it is a male or a female child; he would have done the same because it is his child’), the articulation of these norms by parents suggest that our quantitative findings regarding discriminatory paternal care reflect real behaviour.

In contrast to our finding that fathers bias some care towards sons, our previous research in this population indicates that among recent cohorts parents invest more in their daughters’ education compared with their sons’ (Hedges *et al*. 2018). This may be because, in the context of agropastoralist livelihoods, boys’ subsistence work (farm work, cattle herding) is relatively difficult to combine with school whereas girls’ work (largely domestic tasks) can more easily be done outside of school hours (Hedges *et al*. 2018). Together, these studies highlight that sex-biases in parental care can vary across the child's life course and across the dimension of care considered.

Another tenet of evolutionary parental investment theory is the Trivers–Willard Hypothesis (TWH). This suggests that parents in ‘good condition’ (e.g. resource-rich) will benefit more from investing in offspring of the sex that has greater variation in reproductive success (often males); and parents in ‘poor condition’ (e.g. resource-poor) will benefit more from investing in offspring of the other sex (often females) (Trivers and Willard 1973; Veller *et al*. 2016). However, interpretations of tests of the TWH are been muddled by a widespread failure to first confirm whether the preconditions of the TWH (for details see Trivers and Willard 1973, p. 91) are met. In light of these problems (see Cronk 2007 for a review), and our lack of supporting data to establish these preconditions, we have opted to not test the TWH in this study.

We did however, in supplementary analyses, consider the possibility that sex-biases in care provision may vary by the child's age or birth order, and considered whether alloparenting may compensate for the lack of paternal care provision for girls. To explore child age and birth order we conducted two subsequent analyses. The original regression models for resource allocation, direct/physical care provision and parental marital status and co-habitation were re-run, including an interaction term for a continuous measure of child's age, and including an interaction term for whether the child was their father's first born or not, measured as a binary variable. There was no evidence of an interaction between child's sex and either child's age or child's birth order for any form of care provision (see Supplementary Tables S7.1–S7.17 and S8.1–S8.17). To examine alloparent compensation, we used data on resource allocation and all six forms of direct/physical care provision from five different alloparents (maternal grandparents, paternal grandparents, maternal aunts/uncles, paternal aunts/uncles and child's siblings) collected using the same methods as defined earlier for parents. Logistic regression models tested for associations between each measure of alloparental care and child's sex. We found no evidence of sex-biased care provision from any alloparent (See Supplementary Tables S9.1-S9.2).

## 5. Limitations and future work

Our analysis is limited by some weaknesses inherent in survey-data. For example, it is possible that social desirability bias may have impacted responses to our questions on care provision for children as respondents may be inclined to answer in ways they think others want to hear. However, as our participants were blind to our hypotheses (i.e. not informed that we would compare care of sons with daughters), we consider that this will not have impacted our findings substantially.

It is possible that the extra care sons receive from fathers is surplus and will not impact their survival and eventual reproductive success. If this is the case then a functional/adaptationist perspective on sex-biased parental investment may be misguided. However, the under-5-year age group is a critical period for children and we would expect that even marginal amounts of care could have a potentially significant impact on their wellbeing. Thus, a logical follow-up to this study would be to investigate a link between parental care and children's health and survival.

## 6. Conclusion

We report novel evidence of sex-biased parental care in early childhood among a Sukuma community in northwestern Tanzania. We also add to previous scholarship by providing detailed information on what both fathers and mothers do for their young children in this context. We find that mothers provide more direct/physical care to children, but also observe significant amounts of direct/physical care and resource provisioning from fathers. Furthermore, we find that fathers provide direct/physical care differentially by child's sex while mothers do not discriminate. Sex-biases in fathering appear limited to direct interactive forms of childcare, and are further reflected in local gender norms articulated by parents. An evolutionary perspective predicts that these patterns are ultimately accounted for by higher returns to paternal care in sons over daughters, as has been suggested in past research in other cultural settings (e.g. Nettle 2008). Further research will be required to determine whether or not these patterns are generalizable to related low-income settings, and whether sons actually benefit from more care from their fathers during this vulnerable stage of child development.

## Supplementary material

For supplementary material accompanying this paper visit http://dx.doi.org/10.1017/ehs.2019.14. click here to view supplementary material
