# Supplementary material for: Fathers favour sons, mothers don't discriminate: Sex-biased parental care in northwestern Tanzania
Source: Evol Hum Sci. 2019 Dec 4;1:e13. doi: 10.1017/ehs.2019.14 (PMC10427269; doi:10.1017/ehs.2019.14)
Supplement: Supplementary file 1 [file S2513843X19000148sup001.docx]

**Fathers favour sons, mothers don’t discriminate: Sex-biased parental care in north-western Tanzania**

Supplementary Material

Anushé Hassan, Susan B. Schaffnit, Rebecca Sear, Mark Urassa and David W. Lawson

**Supplementary Table S1:** Bivariate analyses showing correlation between child’s sex and selected child, parent and household-level socio-demographic characteristics: child’s age, if child was father’s first-born, parents residence, household size, number of under-5 children in household, food insecurity, and urban versus rural residence (N=808)

|  | **Girls** | **Boys** | **test-statistic** | **p-value** |
| --- | --- | --- | --- | --- |
| Number of total children 0-5 years | 397 | 411 |  |  |
| Child Characteristics |  |  |  |  |
| **Age in Years - n (%)** |  |  |  |  |
| 0-1 years | 76 (19.14) | 83 (20.19) | Pearson-chi2 (4) = 0.5918 | p=0.964 |
| 1-2 years | 78 (19.65) | 78 (18.98) |  |  |
| 2-3 years | 81 (20.40) | 85 (20.68) |  |  |
| 3-4 years | 94 (23.68) | 90 (21.90) |  |  |
| 4-5 years | 68 (17.13) | 75 (18.25) |  |  |
| **First Child of Biological Father - n (%)** |  |  |  |  |
| Yes | 89 (23.06) | 78 (19.65) | Pearson-chi2 (2) = 1.5356 | p=0.464 |
| No | 291 (75.39) | 314 (79.09) |  |  |
| Don't Know | 6 (1.55) | 5 (1.26) |  |  |
| Parent Characteristics |  |  |  |  |
| **Mother's Residence/Death - n (%)** |  |  |  |  |
| Lives in household | 361 (90.93) | 367 (89.29) | Pearson-chi2 (2) = 1.8254 | p=0.401 |
| Does not live in household | 32 (8.06) | 42 (10.22) |  |  |
| Dead | 4 (1.01) | 2 (0.49) |  |  |
| **Father's Residence/Death - n (%)** |  |  |  |  |
| In the household | 265 (66.75) | 282 (68.61) | Pearson-chi2 (3) = 0.8805 | p=0.830 |
| Not in the household | 123 (30.98) | 117 (28.47) |  |  |
| Dead | 4 (1.01) | 5 (1.22) |  |  |
| Don't Know / Refusal | 5 (1.26) | 7 (1.70) |  |  |
| Household (HH) Characteristics |  |  |  |  |
| **HH size - mean (SD)** | 8.41 (3.6) | 8.42 (3.83) | t-test = -0.0392 | p=0.9687 |
| **Number of 0-5s in HH - mean (SD)** | 2.2 (1.21) | 2.18 (1.23) | t-test = 0.2206 | p=0.8254 |
| **Food Insecurity - mean (SD)** | 10.21 (7.38) | 10.85 (7.73) | t-test = -1.2034 | p=0.2292 |
| **Urban/Rural Residence - n (%)** |  |  |  |  |
| Urban (Town) | 204 (51.39) | 211 (51.34) | Pearson-chi2 (1) = 0.0002 | p=0.989 |
| Rural (Village) | 193 (48.61) | 200 (48.66) |  |  |

**Supplementary Figure S1:** Odds Ratios with 95% Confidence Intervals showing relationship between child’s sex (ref: female) and provisioning of six types of direct/physical care by coresident biological mothers and fathers controlling for age and age-squared (**Mothers N=** Feeding: 728, Playing: 728, Caring if sick: 204, Cosleeping: 727, Supervising: 728, Washing: 728; **Fathers N=** Feeding: 547, Playing: 547, Caring if sick: 143, Cosleeping: 547, Supervising: 547, Washing: 547).

**
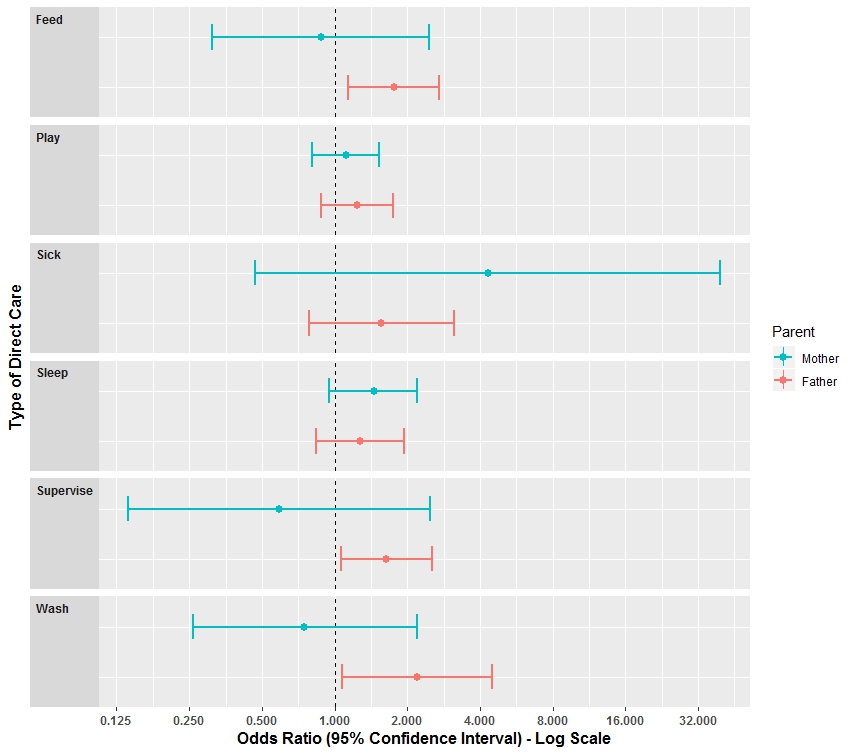
**

**Supplementary Tables 2: Logistic regression models for association between child’s sex and resource provision from all living mothers and non-coresident fathers**

**Supplementary Table S2.1:** Logistic Regression output showing odds of receiving resources from all mothers (excluding children with dead mothers) in the three months preceding the survey for sons versus daughters, controlling for child’s age and age-squared

| Number of obs = 801 |  |  |  |  |  |
| --- | --- | --- | --- | --- | --- |
|  |  |  |  |  |  |
| **Resource Provision -** | **Odds Ratio** | **Std. Err.** | **P>z** | **[95% Conf. Interval]** | |
| **Alive Mothers** |  |  |  |  |  |
|  |  |  |  |  |  |
| Child's Sex |  |  |  |  |  |
| Male | 1.207 | 0.222 | 0.304 | 0.843 | 1.730 |
| Child's Age | 1.338 | 0.340 | 0.252 | 0.813 | 2.203 |
|  |  |  |  |  |  |
| Child's Age-Squared | 0.944 | 0.049 | 0.270 | 0.853 | 1.046 |
|  |  |  |  |  |  |
| _cons | 3.159 | 0.868 | 0.000 | 1.844 | 5.414 |

**Supplementary Table S2.2:** Logistic Regression output showing odds of receiving resources from non-coresident fathers in the three months preceding the survey for sons versus daughters, controlling for child’s age and age-squared

| Number of obs = 239 |  |  |  |  |  |
| --- | --- | --- | --- | --- | --- |
|  |  |  |  |  |  |
| **Resource Provision - Non-Coresident Fathers** | **Odds Ratio** | **Std. Err.** | **P>z** | **[95% Conf. Interval]** | |
|  |  |  |  |  |  |
| Child's Sex |  |  |  |  |  |
| Male | 0.859 | 0.232 | 0.575 | 0.506 | 1.460 |
| Child's Age | 0.739 | 0.277 | 0.419 | 0.354 | 1.539 |
|  |  |  |  |  |  |
| Child's Age-Squared | 0.993 | 0.078 | 0.927 | 0.851 | 1.159 |
|  |  |  |  |  |  |
| _cons | 1.896 | 0.733 | 0.098 | 0.889 | 4.045 |
|  |  |  |  |  |  |

**Supplementary Tables 3: Logistic regression models for association between child’s sex and direct/physical care resource provision from coresident mothers**

**Supplementary Table S3.1:** Logistic Regression output showing odds of coresident mothers washing their sons versus daughters in the 2 weeks preceding the survey, controlling for child’s age and age-squared

| Number of obs = 728 |  |  |  |  |  |
| --- | --- | --- | --- | --- | --- |
|  |  |  |  |  |  |
| **Resident Mother - Washing** | **Odds Ratio** | **Std. Err.** | **P>z** | **[95% Conf.** | **Interval]** |
|  |  |  |  |  |  |
| Child's Sex |  |  |  |  |  |
| Male | 0.751 | 0.411 | 0.600 | 0.257 | 2.192 |
| Child's Age | 0.846 | 0.720 | 0.844 | 0.159 | 4.490 |
|  |  |  |  |  |  |
| Child's Age-Squared | 0.961 | 0.152 | 0.800 | 0.705 | 1.310 |
|  |  |  |  |  |  |
| _cons | 132.936 | 143.360 | 0.000 | 16.058 | 1100.504 |
|  |  |  |  |  |  |

**Supplementary Table S3.2:** Logistic Regression output showing odds of coresident mothers feeding their sons versus daughters in the 2 weeks preceding the survey, controlling for child’s age and age-squared

| Number of obs = 728 |  |  |  |  |  |
| --- | --- | --- | --- | --- | --- |
|  |  |  |  |  |  |
| **Resident Mother - Feeding** | **Odds Ratio** | **Std. Err.** | **P>z** | **[95% Conf.** | **Interval]** |
|  |  |  |  |  |  |
| Child's Sex |  |  |  |  |  |
| Male | 0.879 | 0.461 | 0.805 | 0.314 | 2.458 |
| Child's Age | 0.827 | 0.698 | 0.822 | 0.158 | 4.325 |
|  |  |  |  |  |  |
| Child's Age-Squared | 0.957 | 0.148 | 0.774 | 0.706 | 1.296 |
|  |  |  |  |  |  |
| _cons | 127.606 | 137.713 | 0.000 | 15.390 | 1058.016 |
|  |  |  |  |  |  |

**Supplementary Table S3.3:** Logistic Regression output showing odds of coresident mothers playing with their sons versus daughters in the 2 weeks preceding the survey, controlling for child’s age and age-squared

| Number of obs = 728 |  |  |  |  |  |
| --- | --- | --- | --- | --- | --- |
|  |  |  |  |  |  |
| **Resident Mother - Playing** | **Odds Ratio** | **Std. Err.** | **P>z** | **[95% Conf.** | **Interval]** |
|  |  |  |  |  |  |
| Child's Sex |  |  |  |  |  |
| Male | 1.116 | 0.181 | 0.498 | 0.812 | 1.533 |
| Child's Age | 0.279 | 0.072 | 0.000 | 0.168 | 0.464 |
|  |  |  |  |  |  |
| Child's Age-Squared | 1.214 | 0.061 | 0.000 | 1.099 | 1.340 |
|  |  |  |  |  |  |
| _cons | 9.597 | 2.965 | 0.000 | 5.238 | 17.583 |
|  |  |  |  |  |  |

**Supplementary Table S3.4:** Logistic Regression output showing odds of coresident mothers supervising their sons versus daughters in the 2 weeks preceding the survey, controlling for child’s age and age-squared

| Number of obs = 728 |  |  |  |  |  |
| --- | --- | --- | --- | --- | --- |
|  |  |  |  |  |  |
| **Resident Mother - Supervising** | **Odds Ratio** | **Std. Err.** | **P>z** | **[95% Conf. Interval]** | |
|  |  |  |  |  |  |
| Child's Sex |  |  |  |  |  |
| Male | 0.586 | 0.432 | 0.469 | 0.138 | 2.484 |
| Child's Age | 0.017 | 0.043 | 0.113 | 0.000 | 2.652 |
|  |  |  |  |  |  |
| Child's Age-Squared | 1.890 | 0.804 | 0.135 | 0.821 | 4.349 |
|  |  |  |  |  |  |
| _cons | 40354.870 | 156051.300 | 0.006 | 20.621 | 79000000 |
|  |  |  |  |  |  |

**Supplementary Table S3.5:** Logistic Regression output showing odds of coresident mothers cosleeping with their sons versus daughters in the 2 weeks preceding the survey, controlling for child’s age and age-squared

| Number of obs = 727 |  |  |  |  |  |
| --- | --- | --- | --- | --- | --- |
|  |  |  |  |  |  |
| **Resident Mother - Co-sleeping** | **Odds Ratio** | **Std. Err.** | **P>z** | **[95% Conf.** | **Interval]** |
|  |  |  |  |  |  |
| Child's Sex |  |  |  |  |  |
| Male | 1.445 | 0.310 | 0.086 | 0.949 | 2.201 |
| Child's Age | 0.044 | 0.033 | 0.000 | 0.010 | 0.193 |
|  |  |  |  |  |  |
| Child's Age-Squared | 1.384 | 0.159 | 0.005 | 1.104 | 1.734 |
|  |  |  |  |  |  |
| _cons | 1430.178 | 1697.988 | 0.000 | 139.571 | 14654.95 |
|  |  |  |  |  |  |

**Supplementary Table S3.6:** Logistic Regression output showing odds of coresident mothers caring for their son versus their daughter if they had been sick in the 2 weeks preceding the survey, controlling for child’s age and age-squared

| Number of obs = 204 |  |  |  |  |  |
| --- | --- | --- | --- | --- | --- |
|  |  |  |  |  |  |
| **Resident Mother - Caring if sick** | **Odds Ratio** | **Std. Err.** | **P>z** | **[95% Conf.** | **Interval]** |
|  |  |  |  |  |  |
| Child's Sex |  |  |  |  |  |
| Male | 4.303 | 4.866 | 0.197 | 0.469 | 39.468 |
| Child's Age | 0.079 | 0.180 | 0.267 | 0.001 | 6.962 |
|  |  |  |  |  |  |
| Child's Age-Squared | 1.690 | 0.808 | 0.273 | 0.662 | 4.316 |
|  |  |  |  |  |  |
| _cons | 277.499 | 711.625 | 0.028 | 1.821 | 42276.64 |
|  |  |  |  |  |  |

**Supplementary Tables 4: Logistic regression models for association between child’s sex and direct/physical care resource provision from coresident fathers**

**Supplementary Table S4.1:** Logistic Regression output showing odds of coresident fathers washing their sons versus daughters in the 2 weeks preceding the survey, controlling for child’s age and age-squared

| Number of obs = 547 |  |  |  |  |  |
| --- | --- | --- | --- | --- | --- |
|  |  |  |  |  |  |
| **Resident Father - Washing** | **Odds Ratio** | **Std. Err** | **P>z** | **[95% Conf.** | **Interval]** |
|  |  |  |  |  |  |
| Child's Sex |  |  |  |  |  |
| Male | 2.185 | 0.798 | 0.032 | 1.068 | 4.471 |
| Child's Age | 3.693 | 2.463 | 0.050 | 0.999 | 13.647 |
|  |  |  |  |  |  |
| Child's Age-Squared | 0.830 | 0.100 | 0.121 | 0.655 | 1.051 |
|  |  |  |  |  |  |
| _cons | 0.007 | 0.006 | 0.000 | 0.001 | 0.041 |
|  |  |  |  |  |  |

**Supplementary Table S4.2:** Logistic Regression output showing odds of coresident fathers feeding their sons versus daughters in the 2 weeks preceding the survey, controlling for child’s age and age-squared

| Number of obs = 547 |  |  |  |  |  |
| --- | --- | --- | --- | --- | --- |
|  |  |  |  |  |  |
| **Resident Father - Feeding** | **Odds Ratio** | **Std. Err.** | **P>z** | **[95% Conf.** | **Interval]** |
|  |  |  |  |  |  |
| Child's Sex |  |  |  |  |  |
| Male | 1.759 | 0.389 | 0.011 | 1.139 | 2.714 |
| Child's Age | 2.431 | 0.837 | 0.010 | 1.238 | 4.774 |
|  |  |  |  |  |  |
| Child's Age-Squared | 0.843 | 0.058 | 0.013 | 0.737 | 0.964 |
|  |  |  |  |  |  |
| _cons | 0.076 | 0.032 | 0.000 | 0.034 | 0.172 |
|  |  |  |  |  |  |

**Supplementary Table S4.3:** Logistic Regression output showing odds of coresident fathers playing with their sons versus daughters in the 2 weeks preceding the survey, controlling for child’s age and age-squared

| Number of obs = 547 |  |  |  |  |  |
| --- | --- | --- | --- | --- | --- |
|  |  |  |  |  |  |
| **Resident Father - Playing** | **Odds Ratio** | **Std. Err.** | **P>z** | **[95% Conf.** | **Interval]** |
|  |  |  |  |  |  |
| Child's Sex |  |  |  |  |  |
| Male | 1.238 | 0.216 | 0.220 | 0.880 | 1.742 |
| Child's Age | 0.758 | 0.193 | 0.276 | 0.461 | 1.247 |
|  |  |  |  |  |  |
| Child's Age-Squared | 1.006 | 0.051 | 0.907 | 0.910 | 1.112 |
|  |  |  |  |  |  |
| _cons | 1.791 | 0.520 | 0.045 | 1.014 | 3.163 |
|  |  |  |  |  |  |

**Supplementary Table S4.4:** Logistic Regression output showing odds of coresident fathers supervising their sons versus daughters in the 2 weeks preceding the survey, controlling for child’s age and age-squared

| Number of obs = 547 |  |  |  |  |  |
| --- | --- | --- | --- | --- | --- |
|  |  |  |  |  |  |
| **Resident Father - Supervising** | **Odds Ratio** | **Std. Err.** | **P>z** | **[95% Conf.** | **Interval]** |
|  |  |  |  |  |  |
| Child's Sex |  |  |  |  |  |
| Male | 1.629 | 0.361 | 0.028 | 1.055 | 2.516 |
| Child's Age | 0.759 | 0.245 | 0.393 | 0.404 | 1.428 |
|  |  |  |  |  |  |
| Child's Age-Squared | 1.090 | 0.073 | 0.197 | 0.956 | 1.244 |
|  |  |  |  |  |  |
| _cons | 3.425 | 1.209 | 0.000 | 1.714 | 6.842 |
|  |  |  |  |  |  |

**Supplementary Table S4.5:** Logistic Regression output showing odds of coresident fathers cosleeping with their sons versus daughters in the 2 weeks preceding the survey, controlling for child’s age and age-squared

| Number of obs = 547 |  |  |  |  |  |
| --- | --- | --- | --- | --- | --- |
|  |  |  |  |  |  |
| **Resident Father - Co-sleeping** | **Odds Ratio** | **Std. Err.** | **P>z** | **[95% Conf.** | **Interval]** |
|  |  |  |  |  |  |
| Child's Sex |  |  |  |  |  |
| Male | 1.277 | 0.270 | 0.247 | 0.844 | 1.931 |
| Child's Age | 0.239 | 0.109 | 0.002 | 0.098 | 0.583 |
|  |  |  |  |  |  |
| Child's Age-Squared | 1.097 | 0.087 | 0.243 | 0.939 | 1.280 |
|  |  |  |  |  |  |
| _cons | 46.539 | 29.225 | 0.000 | 13.592 | 159.349 |
|  |  |  |  |  |  |

**Supplementary Table S4.6:** Logistic Regression output showing odds of coresident fathers caring for their son versus their daughter if they had been sick in the 2 weeks preceding the survey, controlling for child’s age and age-squared

| Number of obs = 143 |  |  |  |  |  |
| --- | --- | --- | --- | --- | --- |
|  |  |  |  |  |  |
| **Resident Father - Caring if sick** | **Odds Ratio** | **Std. Err.** | **P>z** | **[95% Conf.** | **Interval]** |
|  |  |  |  |  |  |
| Child's Sex |  |  |  |  |  |
| Male | 1.564 | 0.553 | 0.206 | 0.782 | 3.129 |
| Child's Age | 1.008 | 0.582 | 0.990 | 0.325 | 3.123 |
|  |  |  |  |  |  |
| Child's Age-Squared | 1.057 | 0.129 | 0.653 | 0.831 | 1.343 |
|  |  |  |  |  |  |
| _cons | 0.962 | 0.601 | 0.950 | 0.283 | 3.272 |
|  |  |  |  |  |  |

**Supplementary Tables 5: Association between child’s sex and duration of breastfeeding**

**Supplementary Table S5.1:** Logistic Regression output showing odds of being exclusively breastfed for six months or longer (versus less than six months) for sons compared to daughters, controlling for child’s age and age-squared (n=541)

| Number of obs = 541 |  |  |  |  |  |
| --- | --- | --- | --- | --- | --- |
|  |  |  |  |  |  |
| **Exclusive Breastfeeding** | **Odds Ratio** | **Std. Err.** | **P>z** | **[95% Conf.** | **Interval]** |
|  |  |  |  |  |  |
| Child's Sex |  |  |  |  |  |
| Male | 0.850 | 0.151 | 0.358 | 0.600 | 1.203 |
| Child's Age | 1.097 | 0.639 | 0.874 | 0.350 | 3.438 |
|  |  |  |  |  |  |
| Child's Age-Squared | 0.993 | 0.093 | 0.944 | 0.826 | 1.195 |
|  |  |  |  |  |  |
| _cons | 1.412 | 1.204 | 0.686 | 0.265 | 7.516 |
|  |  |  |  |  |  |

**Supplementary Table S5.2:** Discrete Time Survival Analysis Regression output showing odds of stopping overall breastfeeding as age increases for boys vs girls **for each individual month (1-25).** Age is cut off at 25 months due to data sparsity after this period (i.e. very few children continued breastfeeding after 25 months of age). Results indicate that the odds of stopping breastfeeding increase with child’s age and the highest odds of stopping are at 12 months, 18 months, 20 months and 24 months. There is no evidence of a difference in time at weaning between sons and daughters. *A model including an interaction term between time and child’s sex showed no evidence of a difference and has not been displayed here.*

| Number of obs = 10,808 |  |  |  |  |  |
| --- | --- | --- | --- | --- | --- |
|  |  |  |  |  |  |
| **Event: Stopping Breastfeeding** | **Odds Ratio** | **Std. Err.** | **P>z** | **[95% Conf.** | **Interval** |
|  |  |  |  |  |  |
| **Age at Weaning (months)** |  |  |  |  |  |
| 1 | 0.015 | 0.009 | 0.000 | 0.005 | 0.049 |
| 2 | 1 (empty) | | | | |
| 3 | 0.022 | 0.011 | 0.000 | 0.008 | 0.060 |
| 4 | 0.017 | 0.010 | 0.000 | 0.005 | 0.053 |
| 5 | 0.040 | 0.016 | 0.000 | 0.019 | 0.087 |
| 6 | 0.047 | 0.018 | 0.000 | 0.023 | 0.098 |
| 7 | 0.018 | 0.011 | 0.000 | 0.006 | 0.057 |
| 8 | 0.025 | 0.013 | 0.000 | 0.009 | 0.067 |
| 9 | 0.071 | 0.023 | 0.000 | 0.038 | 0.133 |
| 10 | 0.033 | 0.015 | 0.000 | 0.013 | 0.080 |
| 11 | 0.041 | 0.017 | 0.000 | 0.018 | 0.093 |
| 12 | 1 (base) | | | | |
| 13 | 0.035 | 0.018 | 0.000 | 0.013 | 0.096 |
| 14 | 0.207 | 0.050 | 0.000 | 0.129 | 0.333 |
| 15 | 0.343 | 0.072 | 0.000 | 0.228 | 0.517 |
| 16 | 0.353 | 0.076 | 0.000 | 0.231 | 0.540 |
| 17 | 0.553 | 0.109 | 0.003 | 0.375 | 0.814 |
| 18 | 1.539 | 0.258 | 0.010 | 1.108 | 2.138 |
| 19 | 0.760 | 0.166 | 0.208 | 0.495 | 1.166 |
| 20 | 1.407 | 0.290 | 0.098 | 0.939 | 2.109 |
| 21 | 0.371 | 0.128 | 0.004 | 0.189 | 0.732 |
| 22 | 0.077 | 0.056 | 0.000 | 0.019 | 0.317 |
| 23 | 0.039 | 0.039 | 0.001 | 0.005 | 0.282 |
| 24 | 31.974 | 10.362 | 0.000 | 16.941 | 60.346 |
| 25 | 1 (empty) | | | | |
|  |  |  |  |  |  |
| Child's Sex |  |  |  |  |  |
| Male | 1.103 | 0.110 | 0.326 | 0.907 | 1.341 |
|  |  |  |  |  |  |
| _cons | 0.230 | 0.027 | 0.000 | 0.183 | 0.288 |

**Supplementary Table S5.3:** Discrete Time Event History Analysis Regression output showing odds of stopping overall breastfeeding as age increases for boys vs girls with **age categorised into four groups.** Age is cut off at 25 months due to data sparsity. **Results mirror those in Table S5.2,** and show that the odds of stopping breastfeeding increase as children grow older.

| Number of obs = 11,608 |  |  |  |  |  |
| --- | --- | --- | --- | --- | --- |
|  |  |  |  |  |  |
| **Event: Stopping Breastfeeding** | **Odds Ratio** | **Std. Err.** | **P>z** | **[95% Conf. Interval** | |
|  |  |  |  |  |  |
| **Age at Weaning** |  |  |  |  |  |
| 0-6 months | 1 (base) |  |  |  |  |
| 7-12 months | 7.021 | 1.528 | 0.000 | 4.582 | 10.757 |
| 13-18 months | 17.280 | 3.686 | 0.000 | 11.376 | 26.248 |
| 19-25 months | 49.838 | 10.848 | 0.000 | 32.531 | 76.355 |
|  |  |  |  |  |  |
| Child's Sex |  |  |  |  |  |
| Male | 1.074 | 0.097 | 0.427 | 0.900 | 1.281 |
|  |  |  |  |  |  |
| _cons | 0.005 | 0.001 | 0.000 | 0.004 | 0.008 |

**Supplementary Figure S2:** Hazard of stopping overall breastfeeding for boys and girls (time cut off at 24 months due to data sparsity after this period).

**Supplementary Tables 6: Association between child’s sex and parental marital status and co-habiting status**

**Supplementary Table S6.1:** Logistic Regression output showing odds of parents being married versus divorced if their child was a boy compared to a girl, controlling for age and age-squared, using sample of children whose biological parents were currently or previously married (n=653)

| Number of obs = 653 |  |  |  |  |  |
| --- | --- | --- | --- | --- | --- |
|  |  |  |  |  |  |
| **Parents Married vs Divorced** | **Odds Ratio** | **Std. Err.** | **P>z** | **[95% Conf. Interval]** | |
|  |  |  |  |  |  |
| Child's Sex |  |  |  |  |  |
| Male | 1.002 | 0.222 | 0.994 | 0.649 | 1.546 |
| Child's Age | 1.069 | 0.352 | 0.840 | 0.560 | 2.039 |
|  |  |  |  |  |  |
| Child's Age-Squared | 0.940 | 0.060 | 0.326 | 0.830 | 1.064 |
|  |  |  |  |  |  |
| _cons | 8.218 | 3.240 | 0.000 | 3.795 | 17.797 |
|  |  |  |  |  |  |

**Supplementary Table S6.2:** Logistic Regression output showing odds of parents being married versus divorced if their child was a boy compared to a girl, controlling for age and age-squared, using sample of first-born children only (n=101)

| Number of obs = 101 |  |  |  |  |  |
| --- | --- | --- | --- | --- | --- |
|  |  |  |  |  |  |
| **Parents Married vs Divorced** | **Odds Ratio** | **Std. Err.** | **P>z** | **[95% Conf. Interval]** | |
|  |  |  |  |  |  |
| Child's Sex |  |  |  |  |  |
| Male | 1.131 | 0.528 | 0.792 | 0.453 | 2.822 |
| Child's Age | 4.301 | 2.894 | 0.030 | 1.151 | 16.079 |
|  |  |  |  |  |  |
| Child's Age-Squared | 0.702 | 0.092 | 0.007 | 0.542 | 0.908 |
|  |  |  |  |  |  |
| _cons | 1.136 | 0.842 | 0.863 | 0.266 | 4.857 |
|  |  |  |  |  |  |

**Supplementary Table S6.3:** Logistic Regression output showing odds of child’s biological parents residing with each other versus living apart if their child was a boy compared to a girl, controlling for age and age-squared, using full sample of children (n=793)

| Number of obs = 793 |  |  |  |  |  |
| --- | --- | --- | --- | --- | --- |
|  |  |  |  |  |  |
| **Parents co-habiting vs. not** | **Odds Ratio** | **Std. Err.** | **P>z** | **[95% Conf.** | **Interval]** |
|  |  |  |  |  |  |
| Child's Sex |  |  |  |  |  |
| Male | 1.116 | 0.172 | 0.477 | 0.825 | 1.508 |
| Child's Age | 1.579 | 0.338 | 0.033 | 1.037 | 2.402 |
|  |  |  |  |  |  |
| Child's Age-Squared | 0.912 | 0.040 | 0.035 | 0.837 | 0.994 |
|  |  |  |  |  |  |
| _cons | 1.424 | 0.332 | 0.129 | 0.902 | 2.248 |
|  |  |  |  |  |  |

**Supplementary Table S6.4:** Logistic Regression output showing odds of child’s biological parents residing with each other versus living apart if their child was a boy compared to a girl, controlling for age and age-squared, using sample of first-born children only (n=166)

| Number of obs = 166 |  |  |  |  |  |
| --- | --- | --- | --- | --- | --- |
|  |  |  |  |  |  |
| **Parents co-habiting vs. not** | **Odds Ratio** | **Std. Err.** | **P>z** | **[95% Conf.** | **Interval]** |
|  |  |  |  |  |  |
| Child's Sex |  |  |  |  |  |
| Male | 1.42 | 0.47 | 0.29 | 0.74 | 2.73 |
| Child's Age | 5.54 | 2.77 | 0.00 | 2.08 | 14.75 |
|  |  |  |  |  |  |
| Child's Age-Squared | 0.73 | 0.07 | 0.00 | 0.60 | 0.88 |
|  |  |  |  |  |  |
| _cons | 0.11 | 0.07 | 0.00 | 0.04 | 0.36 |
|  |  |  |  |  |  |

**Supplementary Tables 7: Interaction with child’s age (for all types of care provision)**

**Supplementary Table S7.1:** Logistic Regression output showing odds of child’s mother (all except dead) providing resources to sons versus daughters in the three months preceding the survey with an interaction term for child’s age

| Number of obs = 801 |  |  |  |  |  |  |
| --- | --- | --- | --- | --- | --- | --- |
|  |  |  |  |  |  |  |
| **Resource Provision - All Mothers** | **Odds Ratio** | **Std. Err.** | **z** | **P>z** | **[95%** | **Conf. Interval]** |
|  |  |  |  |  |  |  |
| Child's Sex |  |  |  |  |  |  |
| Female | 1 | (base) |  |  |  |  |
| Male | 1.05 | 0.39 | 0.13 | 0.9 | 0.51 | 2.16 |
|  |  |  |  |  |  |  |
| Child Age | 0.99 | 0.09 | -0.1 | 0.93 | 0.83 | 1.19 |
|  |  |  |  |  |  |  |
| Child’s Sex#Child Age |  |  |  |  |  |  |
| Male | 1.06 | 0.14 | 0.44 | 0.66 | 0.82 | 1.38 |
|  |  |  |  |  |  |  |
| _cons | 4.17 | 1.08 | 5.5 | 0 | 2.51 | 6.93 |

**Supplementary Table S7.2:** Logistic Regression output showing odds of child’s non-coresident fathers providing resources to sons versus daughters in the three months preceding the survey with an interaction term for child’s age

| Number of obs = 239 | |  |  |  |  |  |
| --- | --- | --- | --- | --- | --- | --- |
|  |  |  |  |  |  |  |
| **Resource Provision - Non-Cores Fathers** | **Odds Ratio** | **Std. Err.** | **z** | **P>z** | **[95% Conf. Interval]** | |
|  |  |  |  |  |  |  |
| Child's Sex |  |  |  |  |  |  |
| Female | 1 | (base) |  |  |  |  |
| Male | 0.68 | 0.35 | -0.76 | 0.447 | 0.25 | 1.86 |
|  |  |  |  |  |  |  |
| Child Age | 0.68 | 0.09 | -2.8 | 0.005 | 0.52 | 0.89 |
|  |  |  |  |  |  |  |
| Child’s Sex#Child Age | |  |  |  |  |  |
| Male | 1.11 | 0.21 | 0.54 | 0.588 | 0.76 | 1.61 |
|  |  |  |  |  |  |  |
| _cons | 2.19 | 0.77 | 2.21 | 0.027 | 1.09 | 4.37 |

**Supplementary Table S7.3:** Logistic Regression output showing odds of child’s coresident mother washing sons versus daughters in the 2 weeks preceding the survey with an interaction term for child’s age

| Number of obs = 728 | |  |  |  |  |  |
| --- | --- | --- | --- | --- | --- | --- |
|  |  |  |  |  |  |  |
| **Mother - Washing** | **Odds Ratio** | **Std. Err.** | **z** | **P>z** | **[95%** | **Conf. Interval]** |
|  |  |  |  |  |  |  |
| Child's Sex |  |  |  |  |  |  |
| Female | 1 | (base) |  |  |  |  |
| Male | 3.34 | 4.62 | 0.87 | 0.384 | 0.22 | 50.29 |
|  |  |  |  |  |  |  |
| Child Age | 0.90 | 0.27 | -0.34 | 0.731 | 0.50 | 1.63 |
|  |  |  |  |  |  |  |
| Child’s Sex#Child Age | |  |  |  |  |  |
| Male | 0.60 | 0.26 | -1.18 | 0.239 | 0.26 | 1.40 |
|  |  |  |  |  |  |  |
| _cons | 76.36 | 66.70 | 4.96 | 0 | 13.78 | 423.05 |

**Supplementary Table S7.4:** Logistic Regression output showing odds of child’s coresident mother feeding sons versus daughters in the 2 weeks preceding the survey with an interaction term for child’s age

| Number of obs = 728 |  |  |  |  |  |  |
| --- | --- | --- | --- | --- | --- | --- |
|  |  |  |  |  |  |  |
| **Mother - Feeding** | **Odds Ratio** | **Std. Err.** | **z** | **P>z** | **[95%** | **Conf. Interval]** |
|  |  |  |  |  |  |  |
| Child's Sex |  |  |  |  |  |  |
| Female | 1 | (base) |  |  |  |  |
| Male | 0.06 | 0.10 | -1.63 | 0.103 | 0.00 | 1.77 |
|  |  |  |  |  |  |  |
| Child Age | 0.38 | 0.16 | -2.33 | 0.02 | 0.17 | 0.86 |
|  |  |  |  |  |  |  |
| Child’s Sex#Child Age |  |  |  |  |  |  |
| Male | 2.33 | 1.13 | 1.74 | 0.082 | 0.90 | 6.04 |
|  |  |  |  |  |  |  |
| _cons | 991.55 | 1548.59 | 4.42 | 0 | 46.44 | 21169.69 |

**Supplementary Table S7.5:** Logistic Regression output showing odds of child’s coresident mother playing with sons versus daughters in the 2 weeks preceding the survey with an interaction term for child’s age

| Number of obs = 728 |  |  |  |  |  |  |
| --- | --- | --- | --- | --- | --- | --- |
|  |  |  |  |  |  |  |
| **Mother - Playing** | **Odds Ratio** | **Std. Err.** | **z** | **P>z** | **[95%** | **Conf. Interval]** |
|  |  |  |  |  |  |  |
| Child's Sex |  |  |  |  |  |  |
| Female | 1 | (base) |  |  |  |  |
| Male | 0.66 | 0.23 | -1.23 | 0.22 | 0.33 | 1.29 |
|  |  |  |  |  |  |  |
| Child Age | 0.65 | 0.06 | -4.97 | 0 | 0.54 | 0.77 |
|  |  |  |  |  |  |  |
| Child’s Sex#Child Age |  |  |  |  |  |  |
| Male | 1.24 | 0.15 | 1.77 | 0.077 | 0.98 | 1.57 |
|  |  |  |  |  |  |  |
| _cons | 5.47 | 1.39 | 6.68 | 0 | 3.33 | 9.01 |

**Supplementary Table S7.6:** Logistic Regression output showing odds of child’s coresident mother supervising sons versus daughters in the 2 weeks preceding the survey with an interaction term for child’s age

| Number of obs = 728 | |  |  |  |  |  |
| --- | --- | --- | --- | --- | --- | --- |
|  |  |  |  |  |  |  |
| **Mother - Supervising** | **Odds Ratio** | **Std. Err.** | **z** | **P>z** | **[95%** | **Conf. Interval]** |
|  |  |  |  |  |  |  |
| Child's Sex |  |  |  |  |  |  |
| Female | 1 | (base) |  |  |  |  |
| Male | 4.67 | 8.99 | 0.8 | 0.423 | 0.11 | 203.04 |
|  |  |  |  |  |  |  |
| Child Age | 0.94 | 0.40 | -0.14 | 0.891 | 0.41 | 2.17 |
|  |  |  |  |  |  |  |
| Child’s Sex#Child Age | |  |  |  |  |  |
| Male | 0.51 | 0.30 | -1.15 | 0.249 | 0.16 | 1.61 |
|  |  |  |  |  |  |  |
| _cons | 137.22 | 164.01 | 4.12 | 0 | 13.18 | 1428.20 |

**Supplementary Table S7.7:** Logistic Regression output showing odds of child’s coresident mother cosleeping with sons versus daughters in the 2 weeks preceding the survey with an interaction term for child’s age

| Number of obs = 727 |  |  |  |  |  |  |
| --- | --- | --- | --- | --- | --- | --- |
|  |  |  |  |  |  |  |
| **Mother - Co-sleeping** | **Odds Ratio** | **Std. Err.** | **z** | **P>z** | **[95%** | **Conf. Interval]** |
|  |  |  |  |  |  |  |
| Child's Sex |  |  |  |  |  |  |
| Female | 1 | (base) |  |  |  |  |
| Male | 0.76 | 0.56 | -0.38 | 0.706 | 0.18 | 3.25 |
|  |  |  |  |  |  |  |
| Child Age | 0.30 | 0.05 | -7.83 | 0 | 0.22 | 0.40 |
|  |  |  |  |  |  |  |
| Child’s Sex#Child Age |  |  |  |  |  |  |
| Male | 1.22 | 0.26 | 0.94 | 0.349 | 0.80 | 1.86 |
|  |  |  |  |  |  |  |
| _cons | 122.29 | 65.21 | 9.01 | 0 | 43.00 | 347.79 |

**Supplementary Table S7.8:** Logistic Regression output showing odds of child’s coresident mother caring if sick for sons versus daughters in the 2 weeks preceding the survey with an interaction term for child’s age

| Number of obs = 204 |  |  |  |  |  |  |
| --- | --- | --- | --- | --- | --- | --- |
|  |  |  |  |  |  |  |
| **Mother - Caring if Sick** | **Odds Ratio** | **Std. Err.** | **z** | **P>z** | **[95%** | **Conf. Interval]** |
|  |  |  |  |  |  |  |
| Child's Sex |  |  |  |  |  |  |
| Female | 1 | (base) |  |  |  |  |
| Male | 16.73 | 45.52 | 1.04 | 0.3 | 0.08 | 3459.55 |
|  |  |  |  |  |  |  |
| Child Age | 1.05 | 0.45 | 0.12 | 0.906 | 0.46 | 2.42 |
|  |  |  |  |  |  |  |
| Child’s Sex#Child Age |  |  |  |  |  |  |
| Male | 0.61 | 0.53 | -0.57 | 0.569 | 0.11 | 3.41 |
|  |  |  |  |  |  |  |
| _cons | 20.69 | 21.08 | 2.97 | 0.003 | 2.81 | 152.32 |

**Supplementary Table S7.9:** Logistic Regression output showing odds of child’s coresident father washing sons versus daughters in the 2 weeks preceding the survey with an interaction term for child’s age

| Number of obs = 547 | |  |  |  |  |  |
| --- | --- | --- | --- | --- | --- | --- |
|  |  |  |  |  |  |  |
| **Father - Washing** | **Odds Ratio** | **Std. Err.** | **z** | **P>z** | **[95%** | **Conf. Interval]** |
|  |  |  |  |  |  |  |
| Child's Sex |  |  |  |  |  |  |
| Female | 1 | (base) |  |  |  |  |
| Male | 0.84 | 0.72 | -0.21 | 0.837 | 0.15 | 4.53 |
|  |  |  |  |  |  |  |
| Child Age | 1.10 | 0.25 | 0.44 | 0.66 | 0.71 | 1.71 |
|  |  |  |  |  |  |  |
| Child’s Sex#Child Age | |  |  |  |  |  |
| Male | 1.40 | 0.39 | 1.2 | 0.231 | 0.81 | 2.42 |
|  |  |  |  |  |  |  |
| _cons | 0.04 | 0.02 | -4.94 | 0 | 0.01 | 0.14 |

**Supplementary Table S7.10:** Logistic Regression output showing odds of child’s coresident father feeding sons versus daughters in the 2 weeks preceding the survey with an interaction term for child’s age

| Number of obs = 547 |  |  |  |  |  |  |
| --- | --- | --- | --- | --- | --- | --- |
|  |  |  |  |  |  |  |
| **Father - Feeding** | **Odds Ratio** | **Std. Err.** | **z** | **P>z** | **[95%** | **Conf. Interval]** |
|  |  |  |  |  |  |  |
| Child's Sex |  |  |  |  |  |  |
| Female | 1 | (base) |  |  |  |  |
| Male | 1.53 | 0.72 | 0.92 | 0.36 | 0.61 | 3.82 |
|  |  |  |  |  |  |  |
| Child Age | 1.03 | 0.13 | 0.23 | 0.821 | 0.80 | 1.32 |
|  |  |  |  |  |  |  |
| Child’s Sex#Child Age |  |  |  |  |  |  |
| Male | 1.06 | 0.17 | 0.33 | 0.741 | 0.77 | 1.46 |
|  |  |  |  |  |  |  |
| _cons | 0.17 | 0.06 | -4.82 | 0 | 0.08 | 0.35 |

**Supplementary Table S7.11:** Logistic Regression output showing odds of child’s coresident father playing with sons versus daughters in the 2 weeks preceding the survey with an interaction term for child’s age

| Number of obs = 547 |  |  |  |  |  |  |
| --- | --- | --- | --- | --- | --- | --- |
|  |  |  |  |  |  |  |
| **Father - Playing** | **Odds Ratio** | **Std. Err.** | **z** | **P>z** | **[95%** | **Conf. Interval]** |
|  |  |  |  |  |  |  |
| Child's Sex |  |  |  |  |  |  |
| Female | 1 | (base) |  |  |  |  |
| Male | 0.78 | 0.29 | -0.67 | 0.502 | 0.38 | 1.60 |
|  |  |  |  |  |  |  |
| Child Age | 0.71 | 0.07 | -3.61 | 0 | 0.59 | 0.85 |
|  |  |  |  |  |  |  |
| Child’s Sex#Child Age |  |  |  |  |  |  |
| Male | 1.21 | 0.16 | 1.42 | 0.154 | 0.93 | 1.56 |
|  |  |  |  |  |  |  |
| _cons | 2.24 | 0.61 | 2.95 | 0.003 | 1.31 | 3.82 |

**Supplementary Table S7.12:** Logistic Regression output showing odds of child’s coresident father supervising sons versus daughters in the 2 weeks preceding the survey with an interaction term for child’s age

| Number of obs = 547 | |  |  |  |  |  |
| --- | --- | --- | --- | --- | --- | --- |
|  |  |  |  |  |  |  |
| **Father - Supervising** | **Odds Ratio** | **Std. Err.** | **z** | **P>z** | **[95%** | **Conf. Interval]** |
|  |  |  |  |  |  |  |
| Child's Sex |  |  |  |  |  |  |
| Female | 1 | (base) |  |  |  |  |
| Male | 0.98 | 0.43 | -0.05 | 0.963 | 0.42 | 2.30 |
|  |  |  |  |  |  |  |
| Child Age | 1.03 | 0.11 | 0.27 | 0.784 | 0.83 | 1.28 |
|  |  |  |  |  |  |  |
| Child’s Sex#Child Age | |  |  |  |  |  |
| Male | 1.25 | 0.21 | 1.34 | 0.18 | 0.90 | 1.73 |
|  |  |  |  |  |  |  |
| _cons | 3.17 | 0.98 | 3.72 | 0 | 1.73 | 5.82 |

**Supplementary Table S7.13:** Logistic Regression output showing odds of child’s coresident father cosleeping with sons versus daughters in the 2 weeks preceding the survey with an interaction term for child’s age

| Number of obs = 547 |  |  |  |  |  |  |
| --- | --- | --- | --- | --- | --- | --- |
|  |  |  |  |  |  |  |
| **Father - Co-Sleeping** | **Odds Ratio** | **Std. Err.** | **z** | **P>z** | **[95%** | **Conf. Interval]** |
|  |  |  |  |  |  |  |
| Child's Sex |  |  |  |  |  |  |
| Female | 1 | (base) |  |  |  |  |
| Male | 0.78 | 0.48 | -0.41 | 0.682 | 0.23 | 2.60 |
|  |  |  |  |  |  |  |
| Child Age | 0.37 | 0.05 | -7.12 | 0 | 0.28 | 0.48 |
|  |  |  |  |  |  |  |
| Child’s Sex#Child Age |  |  |  |  |  |  |
| Male | 1.18 | 0.23 | 0.86 | 0.39 | 0.81 | 1.72 |
|  |  |  |  |  |  |  |
| _cons | 33.42 | 15.26 | 7.68 | 0 | 13.65 | 81.79 |

**Supplementary Table S7.14:** Logistic Regression output showing odds of child’s coresident father caring if sick for sons versus daughters in the 2 weeks preceding the survey with an interaction term for child’s age

| Number of obs = 143 |  |  |  |  |  |  |
| --- | --- | --- | --- | --- | --- | --- |
|  |  |  |  |  |  |  |
| **Father - Caring if Sick** | **Odds Ratio** | **Std. Err.** | **z** | **P>z** | **[95%** | **Conf. Interval]** |
|  |  |  |  |  |  |  |
| Child's Sex |  |  |  |  |  |  |
| Female | 1 | (base) |  |  |  |  |
| Male | 0.63 | 0.45 | -0.64 | 0.521 | 0.16 | 2.57 |
|  |  |  |  |  |  |  |
| Child Age | 1.01 | 0.23 | 0.05 | 0.957 | 0.65 | 1.57 |
|  |  |  |  |  |  |  |
| Child’s Sex#Child Age |  |  |  |  |  |  |
| Male | 1.58 | 0.49 | 1.47 | 0.142 | 0.86 | 2.90 |
|  |  |  |  |  |  |  |
| _cons | 1.29 | 0.68 | 0.48 | 0.632 | 0.46 | 3.63 |

**Supplementary Table S7.15:** Logistic Regression output showing odds of child being exclusively breastfed for sons versus daughters, with an interaction term for child’s age

| Number of obs = 541 |  |  |  |  |  |  |
| --- | --- | --- | --- | --- | --- | --- |
|  |  |  |  |  |  |  |
| **Exclusive Breastfeeding** | **Odds Ratio** | **Std. Err.** | **z** | **P>z** | **[95%** | **Conf. Interval]** |
|  |  |  |  |  |  |  |
| Child's Sex |  |  |  |  |  |  |
| Female | 1 | (base) |  |  |  |  |
| Male | 1.77 | 1.05 | 0.96 | 0.339 | 0.55 | 5.69 |
|  |  |  |  |  |  |  |
| Child Age | 1.19 | 0.16 | 1.32 | 0.186 | 0.92 | 1.55 |
|  |  |  |  |  |  |  |
| Child’s Sex#Child Age |  |  |  |  |  |  |
| Male | 0.79 | 0.15 | -1.29 | 0.198 | 0.55 | 1.13 |
|  |  |  |  |  |  |  |
| _cons | 1.02 | 0.44 | 0.04 | 0.968 | 0.44 | 2.35 |

**Supplementary Table S7.16:** Logistic Regression output showing odds of child’s biological parents being married versus divorced if the child was a boy versus a girl with an interaction term for child’s age (sample of children with biological parents who were currently or previously married, n=653)

| Number of obs = 653 |  |  |  |  |  |  |
| --- | --- | --- | --- | --- | --- | --- |
|  |  |  |  |  |  |  |
| **Parents Married vs. Divorced** | **Odds Ratio** | **Std. Err.** | **z** | **P>z** | **[95%** | **Conf. Interval]** |
|  |  |  |  |  |  |  |
| Child's Sex |  |  |  |  |  |  |
| Female | 1 | (base) |  |  |  |  |
| Male | 1.59 | 0.81 | 0.9 | 0.368 | 0.58 | 4.33 |
|  |  |  |  |  |  |  |
| Child Age | 0.85 | 0.10 | -1.38 | 0.168 | 0.68 | 1.07 |
|  |  |  |  |  |  |  |
| Child’s Sex#Child Age |  |  |  |  |  |  |
| Male | 0.85 | 0.14 | -1.01 | 0.31 | 0.61 | 1.17 |
|  |  |  |  |  |  |  |
| _cons | 8.59 | 3.06 | 6.03 | 0 | 4.27 | 17.28 |

**Supplementary Table S7.17:** Logistic Regression output showing odds of child’s biological parents residing with each other if the child was a boy versus a girl with an interaction term for child’s age (n=793)

| Number of obs = 793 | |  |  |  |  |  |
| --- | --- | --- | --- | --- | --- | --- |
|  |  |  |  |  |  |  |
| **Parents co-habiting vs. not** | **Odds Ratio** | **Std. Err.** | **z** | **P>z** | **[95%** | **Conf. Interval]** |
|  |  |  |  |  |  |  |
| Child's Sex |  |  |  |  |  |  |
| Female | 1 | (base) |  |  |  |  |
| Male | 2.13 | 0.66 | 2.42 | 0.015 | 1.16 | 3.92 |
|  |  |  |  |  |  |  |
| Child Age | 1.17 | 0.09 | 1.95 | 0.051 | 1.00 | 1.36 |
|  |  |  |  |  |  |  |
| Child’s Sex#Child Age | |  |  |  |  |  |
| Male | 0.76 | 0.09 | -2.4 | 0.016 | 0.61 | 0.95 |
|  |  |  |  |  |  |  |
| _cons | 1.45 | 0.31 | 1.73 | 0.083 | 0.95 | 2.21 |

**Supplementary Tables 8: Interaction with being father’s first-born child (for all types of care provision)**

**Supplementary Table S8.1:** Logistic Regression output showing odds of child’s mother (all except dead) providing resources to sons versus daughters in the three months preceding the survey (n=766) with an interaction term for child being father’s first born or not

| Number of obs =766 |  |  |  |  |  |  |
| --- | --- | --- | --- | --- | --- | --- |
|  |  |  |  |  |  |  |
| **Resource Provision -** | **Odds Ratio** | **Std. Err.** | **z** | **P>z** | **[95%** | **Conf. Interval]** |
| **All Mothers** |  |  |  |  |  |  |
| Child's Sex |  |  |  |  |  |  |
| Female | 1 | (base) |  |  |  |  |
| Male | 1.38 | 0.31 | 1.5 | 0.147 | 0.89 | 2.14 |
|  |  |  |  |  |  |  |
| Father's First Child |  |  |  |  |  |  |
| No | 1.00 | (base) |  |  |  |  |
| Yes | 0.78 | 0.23 | -0.8 | 0.399 | 0.43 | 1.39 |
|  |  |  |  |  |  |  |
| Child’s Sex#First Child |  |  |  |  |  |  |
| Male#Yes | 0.58 | 0.25 | -1.3 | 0.195 | 0.25 | 1.33 |
|  |  |  |  |  |  |  |
| Child Age | 1.27 | 0.33 | 0.9 | 0.371 | 0.76 | 2.12 |
| Child Age-Squared | 0.96 | 0.05 | -0.7 | 0.474 | 0.87 | 1.07 |
|  |  |  |  |  |  |  |
| _cons | 3.38 | 1.00 | 4.1 | 0 | 1.90 | 6.03 |

**Supplementary Table S8.2:** Logistic Regression output showing odds of non-coresident fathers providing resources to sons versus daughters in the three months preceding the survey (n=228) with an interaction term for child being father’s first born or not

| Number of obs =228 |  |  |  |  |  |  |
| --- | --- | --- | --- | --- | --- | --- |
|  |  |  |  |  |  |  |
| **Resource Provision -** | **Odds Ratio** | **Std. Err.** | **z** | **P>z** | **[95%** | **Conf. Interval]** |
| **Non-Cores Fathers** |  |  |  |  |  |  |
| Child's Sex |  |  |  |  |  |  |
| Female | 1 | (base) |  |  |  |  |
| Male | 1.08 | 0.39 | 0.21 | 0.83 | 0.53 | 2.20 |
|  |  |  |  |  |  |  |
| Father's First Child |  |  |  |  |  |  |
| No | 1.00 | (base) |  |  |  |  |
| Yes | 1.74 | 0.67 | 1.43 | 0.154 | 0.81 | 3.70 |
|  |  |  |  |  |  |  |
| Child’s Sex#First Child |  |  |  |  |  |  |
| Male#Yes | 0.67 | 0.37 | -0.72 | 0.47 | 0.22 | 1.99 |
|  |  |  |  |  |  |  |
| Child Age | 0.78 | 0.31 | -0.62 | 0.535 | 0.36 | 1.69 |
| Child Age-Squared | 0.99 | 0.08 | -0.16 | 0.871 | 0.84 | 1.16 |
|  |  |  |  |  |  |  |
| _cons | 1.38 | 0.63 | 0.71 | 0.475 | 0.57 | 3.37 |

**Supplementary Table S8.3:** Logistic Regression output showing odds of child’s coresident mother washing sons versus daughters in the 2 weeks preceding the survey with an interaction term for child being father’s first born or not

| Number of obs =711 |  |  |  |  |  |  |
| --- | --- | --- | --- | --- | --- | --- |
|  |  |  |  |  |  |  |
| **Mother - Washing** | **Odds Ratio** | **Std. Err.** | **z** | **P>z** | **[95%** | **Conf. Interval]** |
|  |  |  |  |  |  |  |
| Child's Sex |  |  |  |  |  |  |
| Female | 1 | (base) |  |  |  |  |
| Male | 0.86 | 0.53 | -0.3 | 0.802 | 0.26 | 2.85 |
|  |  |  |  |  |  |  |
| Father's First Child |  |  |  |  |  |  |
| No | 1.00 | (base) |  |  |  |  |
| Yes | 1.26 | 1.39 | 0.21 | 0.835 | 0.14 | 11.03 |
|  |  |  |  |  |  |  |
| Child’s Sex#First Child | |  |  |  |  |  |
| Male#Yes | 1.01 | 1.57 | 0 | 0.996 | 0.05 | 21.47 |
|  |  |  |  |  |  |  |
| Child Age | 0.66 | 0.59 | -0.5 | 0.639 | 0.11 | 3.84 |
| Child Age-Squared | 1.02 | 0.17 | 0.14 | 0.891 | 0.73 | 1.43 |
|  |  |  |  |  |  |  |
| _cons | 137.11 | 157.13 | 4.29 | 0 | 14.51 | 1295.81 |

**Supplementary Table S8.4:** Logistic Regression output showing odds of child’s coresident mother feeding sons versus daughters in the 2 weeks preceding the survey with an interaction term for child being father’s first born or not

| Number of obs =572 |  |  |  |  |  |  |
| --- | --- | --- | --- | --- | --- | --- |
|  |  |  |  |  |  |  |
| **Mother - Feeding** | **Odds Ratio** | **Std. Err.** | **z** | **P>z** | **[95%** | **Conf. Interval]** |
|  |  |  |  |  |  |  |
| Child's Sex |  |  |  |  |  |  |
| Female | 1 | (base) |  |  |  |  |
| Male | 0.88 | 0.47 | -0 | 0.814 | 0.31 | 2.48 |
|  |  |  |  |  |  |  |
| Father's First Child |  |  |  |  |  |  |
| No | 1.00 | (base) |  |  |  |  |
| Yes | 1.00 | (empty) |  |  |  |  |
|  |  |  |  |  |  |  |
| Child’s Sex#First Child | |  |  |  |  |  |
| Female#Yes | 1.00 | (empty) |  |  |  |  |
| Male#Yes | 1.00 | (empty) |  |  |  |  |
|  |  |  |  |  |  |  |
| Child Age | 0.93 | 0.79 | -0 | 0.933 | 0.18 | 4.91 |
| Child Age-Squared | 0.93 | 0.15 | -0 | 0.668 | 0.69 | 1.27 |
|  |  |  |  |  |  |  |
| _cons | 89.21 | 96.15 | 4.2 | 0 | 10.79 | 737.69 |

**Supplementary Table S8.5:** Logistic Regression output showing odds of child’s coresident mother playing with sons versus daughters in the 2 weeks preceding the survey with an interaction term for child being father’s first born or not

| Number of obs =711 |  |  |  |  |  |  |
| --- | --- | --- | --- | --- | --- | --- |
|  |  |  |  |  |  |  |
| **Mother - Playing** | **Odds Ratio** | **Std. Err.** | **z** | **P>z** | **[95%** | **Conf. Interval]** |
|  |  |  |  |  |  |  |
| Child's Sex |  |  |  |  |  |  |
| Female | 1 | (base) |  |  |  |  |
| Male | 1.05 | 0.19 | 0.3 | 0.804 | 0.73 | 1.49 |
|  |  |  |  |  |  |  |
| Father's First Child |  |  |  |  |  |  |
| No | 1.00 | (base) |  |  |  |  |
| Yes | 0.98 | 0.28 | -0.1 | 0.945 | 0.56 | 1.70 |
|  |  |  |  |  |  |  |
| Child’s Sex#First Child |  |  |  |  |  |  |
| Male#Yes | 1.84 | 0.82 | 1.4 | 0.171 | 0.77 | 4.40 |
|  |  |  |  |  |  |  |
| Child Age | 0.30 | 0.08 | -4.6 | 0 | 0.18 | 0.50 |
| Child Age-Squared | 1.20 | 0.06 | 3.5 | 0 | 1.08 | 1.32 |
|  |  |  |  |  |  |  |
| _cons | 8.85 | 2.80 | 6.9 | 0 | 4.76 | 16.45 |

**Supplementary Table S8.6:** Logistic Regression output showing odds of child’s coresident mother supervising sons versus daughters in the 2 weeks preceding the survey with an interaction term for child being father’s first born or not

| Number of obs =711 |  |  |  |  |  |  |
| --- | --- | --- | --- | --- | --- | --- |
|  |  |  |  |  |  |  |
| **Mother - Supervising** | **Odds Ratio** | **Std. Err.** | **z** | **P>z** | **[95% Conf. Interval]** | |
|  |  |  |  |  |  |  |
| Child's Sex |  |  |  |  |  |  |
| Female | 1 | (base) |  |  |  |  |
| Male | 0.72 | 0.66 | -0.36 | 0.72 | 0.12 | 4.39 |
|  |  |  |  |  |  |  |
| Father's First Child |  |  |  |  |  |  |
| No | 1.00 | (base) |  |  |  |  |
| Yes | 0.57 | 0.71 | -0.45 | 0.65 | 0.05 | 6.49 |
|  |  |  |  |  |  |  |
| Child’s Sex#First Child | |  |  |  |  |  |
| Male#Yes | 0.74 | 1.26 | -0.18 | 0.86 | 0.03 | 21.29 |
|  |  |  |  |  |  |  |
| Child Age | 0.00 | 0.00 | -1.79 | 0.07 | 0.00 | 2.40 |
| Child Age-Squared | 5.16 | 4.72 | 1.79 | 0.07 | 0.86 | 31.03 |
|  |  |  |  |  |  |  |
| _cons | 35100000 | 263000000 | 2.32 | 0.02 | 14.52 | 84800000000000 |

**Supplementary Table S8.7:** Logistic Regression output showing odds of child’s coresident mother cosleeping with sons versus daughters in the 2 weeks preceding the survey with an interaction term for child being father’s first born or not

| Number of obs =710 |  |  |  |  |  |  |
| --- | --- | --- | --- | --- | --- | --- |
|  |  |  |  |  |  |  |
| **Mother - Co-Sleeping** | **Odds Ratio** | **Std. Err.** | **z** | **P>z** | **[95%** | **Conf. Interval]** |
|  |  |  |  |  |  |  |
| Child's Sex |  |  |  |  |  |  |
| Female | 1 | (base) |  |  |  |  |
| Male | 1.21 | 0.28 | 0.8 | 0.425 | 0.76 | 1.92 |
|  |  |  |  |  |  |  |
| Father's First Child |  |  |  |  |  |  |
| No | 1.00 | (base) |  |  |  |  |
| Yes | 1.24 | 0.49 | 0.56 | 0.576 | 0.58 | 2.68 |
|  |  |  |  |  |  |  |
| Child’s Sex#First Child |  |  |  |  |  |  |
| Male#Yes | 3.72 | 2.60 | 1.88 | 0.06 | 0.95 | 14.64 |
|  |  |  |  |  |  |  |
| Child Age | 0.05 | 0.04 | -3.89 | 0 | 0.01 | 0.23 |
| Child Age-Squared | 1.33 | 0.16 | 2.47 | 0.013 | 1.06 | 1.67 |
|  |  |  |  |  |  |  |
| _cons | 1115.67 | 1315.77 | 5.95 | 0 | 110.58 | 11256.52 |

**Supplementary Table S8.8:** Logistic Regression output showing odds of child’s coresident mother caring if sick for sons versus daughters in the 2 weeks preceding the survey with an interaction term for child being father’s first born or not

| Number of obs =156 |  |  |  |  |  |  |
| --- | --- | --- | --- | --- | --- | --- |
|  |  |  |  |  |  |  |
| **Mother - Caring if Sick** | **Odds Ratio** | **Std. Err.** | **z** | **P>z** | **[95%** | **Conf. Interval]** |
|  |  |  |  |  |  |  |
| Child's Sex |  |  |  |  |  |  |
| Female | 1 | (base) |  |  |  |  |
| Male | 4.75 | 5.38 | 1.4 | 0.169 | 0.52 | 43.78 |
|  |  |  |  |  |  |  |
| Father's First Child |  |  |  |  |  |  |
| No | 1.00 | (base) |  |  |  |  |
| Yes | 1.00 | (empty) |  |  |  |  |
|  |  |  |  |  |  |  |
| Child’s Sex#First Child |  |  |  |  |  |  |
| Female#Yes | 1.00 | (empty) |  |  |  |  |
| Male#Yes | 1.00 | (empty) |  |  |  |  |
|  |  |  |  |  |  |  |
| Child Age | 0.09 | 0.21 | -1 | 0.309 | 0.00 | 9.27 |
| Child Age-Squared | 1.64 | 0.82 | 1 | 0.32 | 0.62 | 4.35 |
|  |  |  |  |  |  |  |
| _cons | 181.05 | 472.93 | 2 | 0.047 | 1.08 | 30283.23 |

**Supplementary Table S8.9:** Logistic Regression output showing odds of child’s coresident father washing sons versus daughters in the 2 weeks preceding the survey with an interaction term for child being father’s first born or not

| Number of obs =543 |  |  |  |  |  |  |
| --- | --- | --- | --- | --- | --- | --- |
|  |  |  |  |  |  |  |
| **Father - Washing** | **Odds Ratio** | **Std. Err.** | **z** | **P>z** | **[95%** | **Conf. Interval]** |
|  |  |  |  |  |  |  |
| Child's Sex |  |  |  |  |  |  |
| Female | 1 | (base) |  |  |  |  |
| Male | 1.79 | 0.77 | 1.36 | 0.175 | 0.77 | 4.16 |
|  |  |  |  |  |  |  |
| Father's First Child |  |  |  |  |  |  |
| No | 1.00 | (base) |  |  |  |  |
| Yes | 0.68 | 0.73 | -0.4 | 0.718 | 0.08 | 5.57 |
|  |  |  |  |  |  |  |
| Child’s Sex#First Child | |  |  |  |  |  |
| Male#Yes | 4.95 | 5.87 | 1.35 | 0.177 | 0.49 | 50.56 |
|  |  |  |  |  |  |  |
| Child Age | 3.73 | 2.62 | 1.88 | 0.061 | 0.94 | 14.75 |
| Child Age-Squared | 0.82 | 0.10 | -1.6 | 0.117 | 0.64 | 1.05 |
|  |  |  |  |  |  |  |
| _cons | 0.01 | 0.01 | -5.3 | 0 | 0.00 | 0.04 |

**Supplementary Table S8.10:** Logistic Regression output showing odds of child’s coresident father feeding sons versus daughters in the 2 weeks preceding the survey with an interaction term for child being father’s first born or not

| Number of obs =543 |  |  |  |  |  |  |
| --- | --- | --- | --- | --- | --- | --- |
|  |  |  |  |  |  |  |
| **Father - Feeding** | **Odds Ratio** | **Std. Err.** | **z** | **P>z** | **[95%** | **Conf. Interval]** |
|  |  |  |  |  |  |  |
| Child's Sex |  |  |  |  |  |  |
| Female | 1 | (base) |  |  |  |  |
| Male | 1.69 | 0.41 | 2.2 | 0.029 | 1.06 | 2.70 |
|  |  |  |  |  |  |  |
| Father's First Child |  |  |  |  |  |  |
| No | 1.00 | (base) |  |  |  |  |
| Yes | 0.68 | 0.38 | -1 | 0.489 | 0.22 | 2.05 |
|  |  |  |  |  |  |  |
| Child’s Sex#First Child | |  |  |  |  |  |
| Male#Yes | 1.64 | 1.15 | 0.7 | 0.484 | 0.41 | 6.50 |
|  |  |  |  |  |  |  |
| Child Age | 2.51 | 0.88 | 2.6 | 0.009 | 1.26 | 5.00 |
| Child Age-Squared | 0.83 | 0.06 | -3 | 0.009 | 0.73 | 0.96 |
|  |  |  |  |  |  |  |
| _cons | 0.08 | 0.03 | -6 | 0 | 0.03 | 0.18 |

**Supplementary Table S8.11:** Logistic Regression output showing odds of child’s coresident father playing with sons versus daughters in the 2 weeks preceding the survey with an interaction term for child being father’s first born or not

| Number of obs =543 |  |  |  |  |  |  |
| --- | --- | --- | --- | --- | --- | --- |
|  |  |  |  |  |  |  |
| **Father - Playing** | **Odds Ratio** | **Std. Err.** | **z** | **P>z** | **[95%** | **Conf. Interval]** |
|  |  |  |  |  |  |  |
| Child's Sex |  |  |  |  |  |  |
| Female | 1 | (base) |  |  |  |  |
| Male | 1.15 | 0.21 | 0.7 | 0.464 | 0.79 | 1.66 |
|  |  |  |  |  |  |  |
| Father's First Child |  |  |  |  |  |  |
| No | 1.00 | (base) |  |  |  |  |
| Yes | 1.12 | 0.42 | 0.3 | 0.76 | 0.54 | 2.33 |
|  |  |  |  |  |  |  |
| Child’s Sex#First Child |  |  |  |  |  |  |
| Male#Yes | 1.96 | 1.06 | 1.2 | 0.213 | 0.68 | 5.66 |
|  |  |  |  |  |  |  |
| Child Age | 0.75 | 0.19 | -1.2 | 0.252 | 0.45 | 1.23 |
| Child Age-Squared | 1.01 | 0.05 | 0.1 | 0.916 | 0.91 | 1.11 |
|  |  |  |  |  |  |  |
| _cons | 1.81 | 0.53 | 2 | 0.043 | 1.02 | 3.23 |

**Supplementary Table S8.12:** Logistic Regression output showing odds of child’s coresident father supervising sons versus daughters in the 2 weeks preceding the survey with an interaction term for child being father’s first born or not

| Number of obs =543 |  |  |  |  |  |  |
| --- | --- | --- | --- | --- | --- | --- |
|  |  |  |  |  |  |  |
| **Father - Supervising** | **Odds Ratio** | **Std. Err.** | **z** | **P>z** | **[95%** | **Conf. Interval]** |
|  |  |  |  |  |  |  |
| Child's Sex |  |  |  |  |  |  |
| Female | 1 | (base) |  |  |  |  |
| Male | 1.59 | 0.37 | 1.98 | 0.05 | 1.00 | 2.52 |
|  |  |  |  |  |  |  |
| Father's First Child |  |  |  |  |  |  |
| No | 1.00 | (base) |  |  |  |  |
| Yes | 1.20 | 0.55 | 0.4 | 0.69 | 0.49 | 2.93 |
|  |  |  |  |  |  |  |
| Child’s Sex#First Child | |  |  |  |  |  |
| Male#Yes | 1.24 | 0.89 | 0.29 | 0.77 | 0.30 | 5.08 |
|  |  |  |  |  |  |  |
| Child Age | 0.75 | 0.24 | -0.9 | 0.37 | 0.40 | 1.41 |
| Child Age-Squared | 1.09 | 0.07 | 1.31 | 0.19 | 0.96 | 1.25 |
|  |  |  |  |  |  |  |
| _cons | 3.41 | 1.21 | 3.45 | 0 | 1.70 | 6.86 |

**Supplementary Table S8.13:** Logistic Regression output showing odds of child’s coresident father cosleeping with sons versus daughters in the 2 weeks preceding the survey with an interaction term for child being father’s first born or not

| Number of obs =543 |  |  |  |  |  |  |
| --- | --- | --- | --- | --- | --- | --- |
|  |  |  |  |  |  |  |
| **Father - Co-Sleeping** | **Odds Ratio** | **Std. Err.** | **z** | **P>z** | **[95%** | **Conf. Interval]** |
|  |  |  |  |  |  |  |
| Child's Sex |  |  |  |  |  |  |
| Female | 1 | (base) |  |  |  |  |
| Male | 1.23 | 0.28 | 0.91 | 0.361 | 0.79 | 1.92 |
|  |  |  |  |  |  |  |
| Father's First Child |  |  |  |  |  |  |
| No | 1.00 | (base) |  |  |  |  |
| Yes | 1.27 | 0.56 | 0.55 | 0.585 | 0.54 | 3.02 |
|  |  |  |  |  |  |  |
| Child’s Sex#First Child |  |  |  |  |  |  |
| Male#Yes | 1.58 | 1.04 | 0.69 | 0.487 | 0.43 | 5.73 |
|  |  |  |  |  |  |  |
| Child Age | 0.24 | 0.11 | -3.1 | 0.002 | 0.10 | 0.59 |
| Child Age-Squared | 1.09 | 0.09 | 1.12 | 0.262 | 0.94 | 1.28 |
|  |  |  |  |  |  |  |
| _cons | 45.52 | 28.68 | 6.06 | 0 | 13.24 | 156.48 |

**Supplementary Table S8.14:** Logistic Regression output showing odds of child’s coresident father caring if sick for sons versus daughters in the 2 weeks preceding the survey with an interaction term for child being father’s first born or not

| Number of obs =140 |  |  |  |  |  |  |
| --- | --- | --- | --- | --- | --- | --- |
|  |  |  |  |  |  |  |
| **Father - Caring if Sick** | **Odds Ratio** | **Std. Err.** | **z** | **P>z** | **[95%** | **Conf. Interval]** |
|  |  |  |  |  |  |  |
| Child's Sex |  |  |  |  |  |  |
| Female | 1 | (base) |  |  |  |  |
| Male | 1.60 | 0.61 | 1.2 | 0.22 | 0.76 | 3.37 |
|  |  |  |  |  |  |  |
| Father's First Child |  |  |  |  |  |  |
| No | 1.00 | (base) |  |  |  |  |
| Yes | 1.94 | 1.46 | 0.9 | 0.378 | 0.44 | 8.47 |
|  |  |  |  |  |  |  |
| Child’s Sex#First Child |  |  |  |  |  |  |
| Male#Yes | 2.30 | 3.06 | 0.6 | 0.533 | 0.17 | 31.36 |
|  |  |  |  |  |  |  |
| Child Age | 1.22 | 0.73 | 0.3 | 0.741 | 0.38 | 3.96 |
| Child Age-Squared | 1.02 | 0.13 | 0.1 | 0.902 | 0.79 | 1.31 |
|  |  |  |  |  |  |  |
| _cons | 0.72 | 0.47 | -0.5 | 0.612 | 0.20 | 2.60 |

**Supplementary Table S8.15:** Logistic Regression output showing odds of child being exclusively breastfed for sons versus daughters, with an interaction term for child being father’s first born or not

| Number of obs =515 |  |  |  |  |  |  |
| --- | --- | --- | --- | --- | --- | --- |
|  |  |  |  |  |  |  |
| **Exclusive Breastfeeding** | **Odds Ratio** | **Std. Err.** | **z** | **P>z** | **[95%** | **Conf. Interval]** |
|  |  |  |  |  |  |  |
| Child's Sex |  |  |  |  |  |  |
| Female | 1 | (base) |  |  |  |  |
| Male | 0.70 | 0.14 | -1.7 | 0.081 | 0.46 | 1.05 |
|  |  |  |  |  |  |  |
| Father's First Child |  |  |  |  |  |  |
| No | 1.00 | (base) |  |  |  |  |
| Yes | 0.53 | 0.16 | -2 | 0.042 | 0.29 | 0.98 |
|  |  |  |  |  |  |  |
| Child’s Sex#First Child |  |  |  |  |  |  |
| Male#Yes | 3.12 | 1.41 | 2.5 | 0.012 | 1.28 | 7.58 |
|  |  |  |  |  |  |  |
| Child Age | 0.94 | 0.59 | -0.1 | 0.923 | 0.28 | 3.20 |
| Child Age-Squared | 1.02 | 0.10 | 0.2 | 0.874 | 0.83 | 1.24 |
|  |  |  |  |  |  |  |
| _cons | 2.07 | 1.90 | 0.8 | 0.431 | 0.34 | 12.58 |

**Supplementary Table S8.16:** Logistic Regression output showing odds of. child’s biological parents being married versus divorced if the child was a boy versus a girl with an interaction term for child being father’s first born or not (sample of children with biological parents who were currently or previously married, n=649)

| Number of obs =649 |  |  |  |  |  |  |
| --- | --- | --- | --- | --- | --- | --- |
|  |  |  |  |  |  |  |
| **Parents Married vs. Divorced** | **Odds Ratio** | **Std. Err.** | **z** | **P>z** | **[95%** | **Conf. Interval]** |
|  |  |  |  |  |  |  |
| Child's Sex |  |  |  |  |  |  |
| Female | 1 | (base) |  |  |  |  |
| Male | 1.07 | 0.29 | 0.26 | 0.793 | 0.63 | 1.83 |
|  |  |  |  |  |  |  |
| Father's First Child |  |  |  |  |  |  |
| No | 1.00 | (base) |  |  |  |  |
| Yes | 0.31 | 0.11 | -3.33 | 0.001 | 0.15 | 0.61 |
|  |  |  |  |  |  |  |
| Child’s Sex#First Child |  |  |  |  |  |  |
| Male#Yes | 0.85 | 0.44 | -0.32 | 0.751 | 0.31 | 2.32 |
|  |  |  |  |  |  |  |
| Child Age | 0.91 | 0.32 | -0.26 | 0.797 | 0.46 | 1.81 |
| Child Age-Squared | 0.98 | 0.07 | -0.37 | 0.709 | 0.86 | 1.11 |
|  |  |  |  |  |  |  |
| _cons | 11.78 | 5.04 | 5.77 | 0 | 5.10 | 27.25 |

**Supplementary Table S8.17:** Logistic Regression output showing odds of child’s biological parents residing with each other if the child was a boy versus a girl with an interaction term for child being father’s first born or not (n=767)

| Number of obs =767 |  |  |  |  |  |  |
| --- | --- | --- | --- | --- | --- | --- |
|  |  |  |  |  |  |  |
| **Parents co-habiting vs. not** | **Odds Ratio** | **Std. Err.** | **z** | **P>z** | **[95%** | **Conf. Interval]** |
|  |  |  |  |  |  |  |
| Child's Sex |  |  |  |  |  |  |
| Female | 1 | (base) |  |  |  |  |
| Male | 0.98 | 0.20 | -0.1 | 0.93 | 0.66 | 1.45 |
|  |  |  |  |  |  |  |
| Father's First Child |  |  |  |  |  |  |
| No | 1.00 | (base) |  |  |  |  |
| Yes | 0.17 | 0.04 | -6.8 | 0 | 0.10 | 0.28 |
|  |  |  |  |  |  |  |
| Child’s Sex#First Child | |  |  |  |  |  |
| Male#Yes | 1.34 | 0.50 | 0.77 | 0.439 | 0.64 | 2.79 |
|  |  |  |  |  |  |  |
| Child Age | 1.45 | 0.34 | 1.6 | 0.11 | 0.92 | 2.30 |
| Child Age-Squared | 0.93 | 0.04 | -1.5 | 0.136 | 0.85 | 1.02 |
|  |  |  |  |  |  |  |
| _cons | 2.63 | 0.71 | 3.6 | 0 | 1.55 | 4.45 |

**Supplementary Tables 9: Additional analyses exploring sex-biased care provision from a number of different alloparents**

**Supplementary Table S9.1:** Logistic regression outputs showing associations between child’s sex and provision of material resources from five different alloparents (maternal grandparents, paternal grandparents, maternal aunts/uncles, paternal aunts/uncles and child’s siblings). Effect sizes (Odds Ratios) adjusted for child’s age (continuous) and age-squared.

|  | Odds Ratio  (95% CI) | | | | |
| --- | --- | --- | --- | --- | --- |
| **Alloparent** | **Maternal Grandparent** | **Paternal Grandparent** | **Maternal Aunt/Uncle** | **Paternal Aunt/Uncle** | **Child's Sibling** |
|  | **Resource Provision** | | | | |
| n | 808 | 808 | 808 | 808 | 808 |
| Child is Male | 0.86  (0.62-1.20) | 1.25  (0.81-1.93) | 1.09  (0.70-1.73) | 1.01  (0.57-1.81) | 1.10  (0.54-2.23) |
| Child's Age | 0.72  (0.46-1.15) | 1.44  (0.77-2.69) | 0.88  (0.47-1.67) | 2.74*  (1.05-7.18) | 0.80  (0.29-2.15) |
| Child's Age-squared | 1.08  (0.98-1.18) | 0.89~  (0.78-1.02) | 1.02  (0.90-1.16 | 0.82  (0.68-0.99) | 1.06  (0.97-1.30) |
| ~p<0.10; *p<0.05; **p<0.01; ***p<0.001 | | | | | |

**Supplementary Table S9.2:** Logistic regression outputs showing associations between child’s sex and each type of direct/physical care provision from five different alloparents (maternal grandparents, paternal grandparents, maternal aunts/uncles, paternal aunts/uncles and child’s siblings). Effect sizes (Odds Ratios) adjusted for child’s age (continuous) and age-squared. Caring for sick children is limited to children who had been sick in past two weeks (n=215).

|  | Odds Ratio (95% CI) | | | | |
| --- | --- | --- | --- | --- | --- |
| **Alloparent** | **Maternal Grandparent** | **Paternal Grandparent** | **Maternal Aunt/Uncle** | **Paternal Aunt/Uncle** | **Child's**  **Sibling** |
|  | **Washing** | | | | |
| n | 808 | 808 | 808 | 808 | 808 |
| Child is Male | 0 .91 (0.65-1.28) | 1.13  (0.72-1.77) | 1.08  (0.74-1.57) | 1.00  (0.60-1.68) | 1.28~  (0.96-1.71) |
| Child's Age | 0.79  (0.49-1.28) | 1.99*  (1.00-3.93) | 1.43  (0.81-2.54) | 2.00~  (0.89-4.49) | 2.47***  (1.59-3.85) |
| Child's Age-squared | 1.06 (0.96-1.17) | 0.84*  (0.72-0.97) | 0.95  (0.85-1.07) | 0.87~  (0.74-1.02) | 0.86**  (0.79-0.94) |
|  | **Feeding** | | | | |
| n | 808 | 808 | 808 | 808 | 808 |
| Child is Male | 1.04 (0.73-1.48) | 1.54~  (0.97-2.44) | 1.25  (0.84-1.84) | 1.11  (0.67-1.86) | 1.29~  (0.96-1.74) |
| Child's Age | 1.53 (0.90-2.60) | 4.94***  (2.20-11.10) | 1.65  (0.90-3.01) | 3.99**  (1.60-9.94) | 3.22***  (2.02-5.12) |
| Child's Age-squared | 0.95 (0.86-1.06) | 0.71***  (0.60-0.84) | 0.93  (0.83-1.05) | 0.76  (0.64-0.92) | 0.83***  (0.75-0.90) |
| ~p<0.10; *p<0.05; **p<0.01; ***p<0.001 | | |  |  |  |
|  | Odds Ratio (95% CI) | | | | |
| **Alloparent** | **Maternal Grandparent** | **Paternal Grandparent** | **Maternal Aunt/Uncle** | **Paternal Aunt/Uncle** | **Child's**  **Sibling** |
|  | **Playing** | | | | |
| n | 808 | 808 | 807 | 807 | 808 |
| Child is Male | 1.11  (0.73-1.68) | 1.58~  (0.98-2.56) | 1.44~  (0.97-2.15) | 1.09  (0.62-1.90) | 1.12  (0.81-1.57) |
| Child's Age | 0.41**  (0.23-0.71) | 1.06  (0.55-2.06) | 0.77  (0.44-1.33) | 1.03  (0.47-2.25) | 3.05***  (1.95-4.77) |
| Child's Age-squared | 1.17**  (1.04-1.31) | 0.94  (0.81-1.08) | 1.05  (0.94-1.18) | 0.92  (0.77-1.10) | 0.84***  (0.77-0.92) |
|  | **Supervising** | | | | |
| n | 808 | 808 | 808 | 807 | 808 |
| Child is Male | 0.96  (0.70-1.33) | 1.22  (0.81-1.83) | 1.23  (0.85-1.78) | 1.17  (0.68-2.01) | 0.98  (0.74-1.31) |
| Child's Age | 0.72  (0.46-1.12) | 1.63  (0.89-2.96) | 0.82  (0.49-1.37) | 1.38  (0.62-3.08) | 1.82**  (1.19-2.79) |
| Child's Age-squared | 1.08~  (0.99-1.18) | 0.87*  (0.77-0.99) | 1.05  (0.94-1.16) | 0.94  (0.80-1.10) | 0.90*  (0.83-0.98) |
| ~p<0.10; *p<0.05; **p<0.01; ***p<0.001 | | |  |  |  |

|  | Odds Ratio (95% CI) | | | | |
| --- | --- | --- | --- | --- | --- |
| **Alloparent** | **Maternal Grandparent** | **Paternal Grandparent** | **Maternal Aunt/Uncle** | **Paternal Aunt/Uncle** | **Child's**  **Sibling** |
|  | **Caring if Sick** | | | | |
| n | 215 | 215 | 215 | 215 | 215 |
| Child is Male | 0.95  (0.49-1.82) | 0.57  (0.20-1.63) | 3.34*  (1.03-10.79) | 0.57  (1.13-2.48) | 0.70  (0.34-1.45) |
| Child's Age | 0.77  (0.28-2.11) | 2.47  (0.39-15.62) | 4.07  (0.54-30.68) | 1.75  (0.15-20.48) | 3.93~  (0.99-15.57) |
| Child's Age-squared | 1.09  (0.89-1.33) | 0.80  (0.54-1.20) | 0.83  (0.57-1.19) | 0.83  (0.47-1.46) | 0.82  (0.64-1.07) |
|  | **Co-Sleeping** | | | | |
| n | 807 | 808 | 808 | 808 | 808 |
| Child is Male | 0.72  (0.43-1.20) | 1.76  (0.73-4.27) | 1.48  (0.74-2.96) | 0.48  (0.09-2.65) | 1.00  (0.71-1.42) |
| Child's Age | 2.63*  (1.04-6.64) | 32.41*  (2.14-491.84) | 1.03  (0.35-3.03) | 2.16  (0.08-56.88) | 2.56**  (1.32-4.96) |
| Child's Age-squared | 0.89  (0.75-1.06) | 0.58*  (0.36-0.91) | 1.07  (0.87-1.31) | 0.96  (0.55-1.69) | 0.96  (0.85-1.08) |
| ~p<0.10; *p<0.05; **p<0.01; ***p<0.001 | | |  |  |  |
